# Chromosomal Translocations in the Parasite *Leishmania* by a MRE11/RAD50-Independent Microhomology-Mediated End Joining Mechanism

**DOI:** 10.1371/journal.pgen.1006117

**Published:** 2016-06-17

**Authors:** Marie-Claude N. Laffitte, Philippe Leprohon, Maripier Hainse, Danielle Légaré, Jean-Yves Masson, Marc Ouellette

**Affiliations:** 1 Centre de Recherche en Infectiologie, CRCHU de Québec, Québec City, Québec, Canada; 2 Genome Stability Laboratory, CRCHU de Québec, Pavillon HDQ Oncology axis, Québec City, Québec, Canada; 3 Department of Molecular Biology, Medical Biochemistry and Pathology, Centre de recherche sur le Cancer, Université Laval, Québec City, Québec, Canada; Duke University, UNITED STATES

## Abstract

The parasite *Leishmania* often relies on gene rearrangements to survive stressful environments. However, safeguarding a minimum level of genome integrity is important for cell survival. We hypothesized that maintenance of genomic integrity in *Leishmania* would imply a leading role of the MRE11 and RAD50 proteins considering their role in DNA repair, chromosomal organization and protection of chromosomes ends in other organisms. Attempts to generate *RAD50* null mutants in a wild-type background failed and we provide evidence that this gene is essential. Remarkably, inactivation of *RAD50* was possible in a *MRE11* null mutant that we had previously generated, providing good evidence that *RAD50* may be dispensable in the absence of *MRE11*. Inactivation of the *MRE11* and *RAD50* genes led to a decreased frequency of homologous recombination and analysis of the null mutants by whole genome sequencing revealed several chromosomal translocations. Sequencing of the junction between translocated chromosomes highlighted microhomology sequences at the level of breakpoint regions. Sequencing data also showed a decreased coverage at subtelomeric locations in many chromosomes in the *MRE11*^*-/-*^*RAD50*^*-/-*^ parasites. This study demonstrates an MRE11-independent microhomology-mediated end-joining mechanism and a prominent role for MRE11 and RAD50 in the maintenance of genomic integrity. Moreover, we suggest the possible involvement of RAD50 in subtelomeric regions stability.

## Author Summary

The parasite *Leishmania* relies on gene rearrangements to survive stressful conditions. However, maintaining a minimum level of genomic integrity is crucial for cell survival. Studies in other organisms have provided evidence that the DNA repair proteins MRE11 and RAD50 are involved in chromosomes organization, protection of chromosomes ends and therefore in the maintenance of genomic integrity. In this manuscript, we present the conditional inactivation of the *Leishmania infantum RAD50* gene that was only possible in *MRE11* deficient cells and suggest the genetic background is crucial for *RAD50* inactivation. We demonstrate the occurrence of chromosomal translocations in the MRE11 and RAD50 deficient cells and described a MRE11-independent microhomology-mediated end-joining mechanism at the level of translocation breakpoints. We also suggest a possible involvement of RAD50 in subtelomeric regions stability. Our results highlight that both MRE11 and RAD50 are important for the maintenance of genomic integrity in *Leishmania*.

## Introduction

Genomic integrity maintenance is essential for cellular development and viability [1–3]. Failure to repair DNA will lead to genomic instability (reviewed in [4–7]). DNA structural changes can manifest as inversion, deletion, duplication, translocation, chromosome end-to-end fusion, aneuploidy [8–10] and some of these events such as gene amplification have been associated in *Leishmania* with response to drug and oxidative stress [11–14]. Increased numbers of DNA rearrangements have been reported in many inherited cancer susceptibility human syndromes [9]. Specific DNA repair genes are mutated in these genomic disorders such as ATM in the Ataxia telangiectasia syndrome, MRE11 in the Ataxia telangiectasia-like disorder, NBS1 in the Nijmegen breakage syndrome and BLM in the Bloom’s syndrome [15–18]. It has been suggested that errors occurring during DNA replication such as stalled or broken replication forks can lead, if left unrepaired, to DNA double strand breaks (DSBs) that are precursors of DNA rearrangements [10,19]. DSBs can also occur during replication or result from exposure to DNA-damaging agents such as ionizing radiation or chemotherapeutic drugs [1,20]. The two main strategies to cope with DSBs are non-homologous end joining (NHEJ) and homologous recombination (HR) [20]. However, only a few NHEJ factors are present in *Leishmania* (MRE11, Ku70/Ku80 and APTX) while Artemis, XRCC4 and the DNA ligase IV are absent, suggesting that this pathway is not functional in the parasite [21–23]. Another pathway that is normally suppressed when NHEJ is present is called microhomology-mediated end joining (MMEJ) or alternative end joining and has been reported in the related parasite *Trypanosoma brucei* [22–24]. In MMEJ, small regions of homology (2 to 20 nucleotides) are used for ligation after resection of each DNA ends in a Ku-independent manner [20]. In this process, DNA ends created from DSBs are recognized by PARP-1 and resected by the MRN (MRE11-RAD50-NBS1) complex followed by annealing and ligation of the two ends by XRCC1/DNA ligase III [25,26]. The HR pathway has been shown to be important for the recovery of stalled replication forks, genomic integrity and telomere maintenance [27–29]. In HR, DSBs are first recognized by the MRN complex and resected by EXO1 and MRE11. Therefore, MMEJ and HR share the same initial step of resection which involves the MRN complex in order to produce regions of homology. Nevertheless, the length of DNA resection as well as the length of the homologous sequence differ between the two processes [23,30]. The tripartite MRN complex has been shown to act as DSBs sensor and DSBs repair effector and is also associated with telomere maintenance [31–33], displaying a major role in the maintenance of genomic stability [34–37]. The complex is composed of MRE11 and RAD50, highly conserved between species, and NBS1 (also represented by XRS2 in yeast) is less conserved and only present in eukaryotes. We previously demonstrated that LiMRE11 displays the same DNA binding and exonuclease activity as human MRE11, but the protein is not essential in *Leishmania infantum* [38]. In addition, we showed the importance of MRE11 and its nuclease domain in extrachromosomal linear amplicons formation under drug pressure. The RAD50 protein is a DNA binding ATPase that displays sequence and structural homology to structural maintenance of chromosome (SMC) family members. An anti-parallel coiled-coil domain contains a central zinc hook (CXXC) motif and might contribute in holding together separate DNA ends [8,34]. The third member of the complex is NBS1 and possess a MRE11 binding domain. NBS1 is thought to stimulate the MRE11-RAD50 complex DNA binding and nuclease activities but biochemical activities of the NBS1 protein itself are not yet elucidated [8,34]. Disruption of the *MRE11* and *RAD50* genes have been shown to increase gene rearrangements rate up to 1000 fold [2,39]. Null mutations in any of the MRN proteins lead to embryonic lethality in mice and have been associated in yeast with DNA rearrangements and chromosome loss events as well as defect in both HR and NHEJ [39–44]. In this manuscript, we present the conditional inactivation of *L*. *infantum RAD50* orthologue, a gene essential in the MRE11 proficient wild-type background but apparently dispensable in the *MRE11*^*-/-*^ background. We also demonstrate chromosomal translocations in the MRE11 and RAD50 deficient cells. These translocations happened through a MRE11-independent MMEJ mechanism where sequence microhomology were found at the translocations breakpoints.

## Results

### Attempts for inactivation of the *Leishmania infantum RAD50* gene

**Fig 1:**
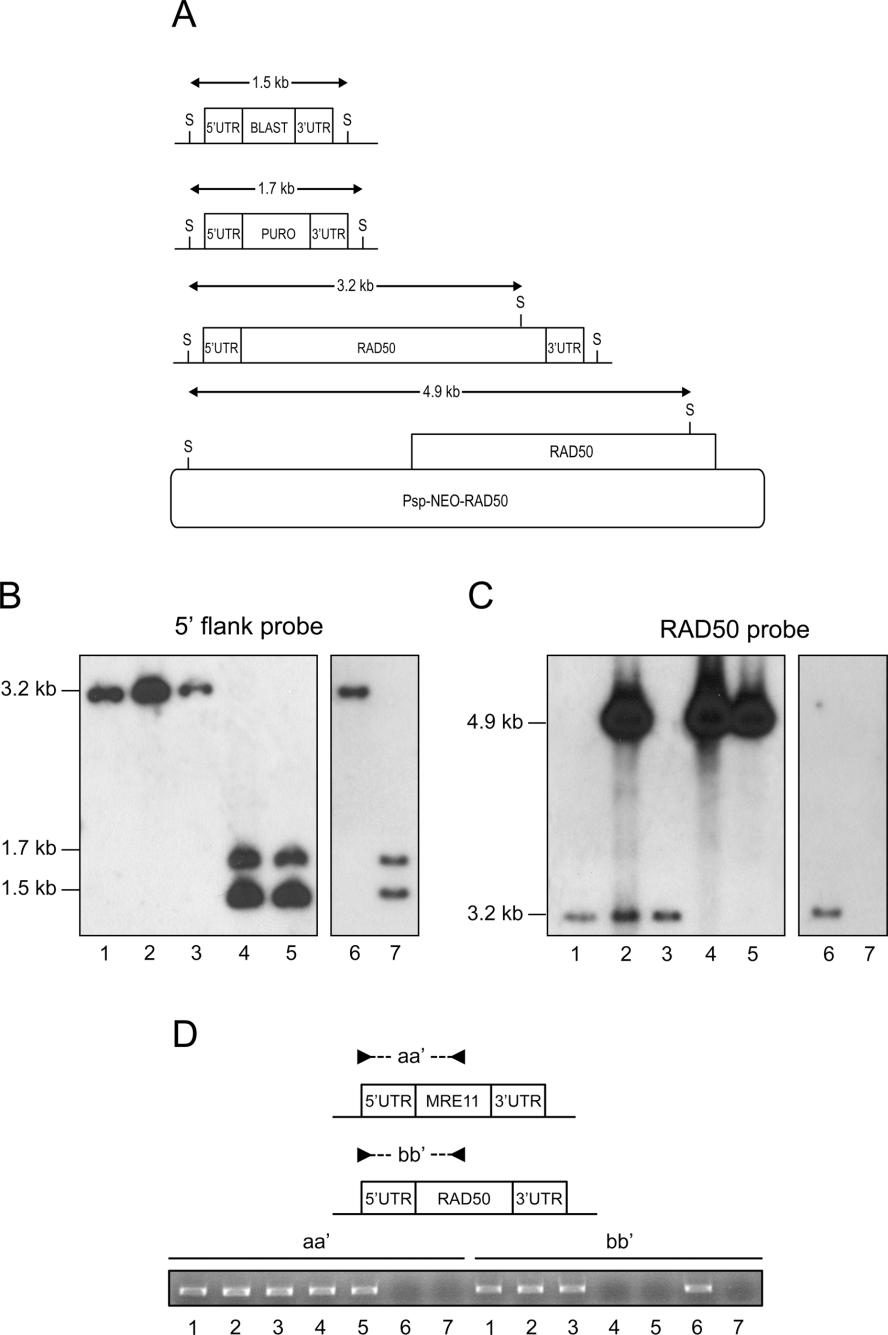
*RAD50* gene conditional inactivation in *L*. *infantum*. **(A)** Schematic representation of the *RAD50* locus in *L*. *infantum* before and after integration of the inactivation cassettes blasticidin-S deaminase (5’-*BLAST-3’*), puromycin acetyltransferase (5’-*PURO-3’*) and transfection construct Psp-*NEO*-*RAD50*. S, SacI restriction sites. **(B, C)** Southern blot analysis with genomic DNAs digested with SacI were hybridized with probes covering either the 5’ flanking region of *RAD50* **(B)** or the *RAD50* ORF **(C). (D)** PCR analysis with primers set aa’ and bb’ the chromosomal copies of the *MRE11* and *RAD50* genes respectively. Lanes: 1, *L*.*infantum* WT; 2, WT Psp-*NEO-RAD50*; 3, WT Rev Psp-*NEO*-*RAD50*; 4, *RAD50*^*-/-*^ Psp-*NEO*-*RAD50*; 5, *RAD50*^*-/-*^ Rev Psp-*NEO*-*RAD50* grown for 55 passages in absence of G418; 6, *MRE11*^*-/-*^ and 7, *MRE11*^*-/-*^*RAD50*^*-/-*^.

It is standard practice to generate null mutant parasites by replacing the entire ORF with resistance markers. The genes coding for the blasticidin-S deaminase (*BLAST*) and puromycin acetyltransferase (*PURO*) were cloned between the 5’- and 3’- *L*. *infantum RAD50* flanking regions and the *BLAST* or *PURO* constructs were transfected independently by electroporation. Hybridization with a 5’UTR probe should lead to 3.2, 1.7 and 1.5 kb SacI-SacI bands in the wild-type (WT), *BLAST RAD50*^*-/+*^, *PURO RAD50*^*-/+*^ cells respectively (Fig 1A). We generated *BLAST RAD50*^*-/+*^ and *PURO RAD50*^*-/+*^ heterozygous lines (S1B Fig, lanes 2–3), but surprisingly, in the *BLAST*/*PURO*/WT *RAD50*^*-/-/+*^ line we observed a remaining intact *RAD50* allele (S1B Fig, lane 4). Despite many attempts, the generation of a *RAD50* null mutant failed. This generation of polyploidy at specific locus is frequently observed in *Leishmania* [45–47] and is thought to occur at locus reputed to be essential. To provide further support for the essentiality of *RAD50*, we first introduced a *RAD50* rescue plasmid (Psp-*NEO*-*RAD50*^*WT*^, Fig 1A) in the *BLAST RAD50*^*-/+*^ cells. Upon the transfection of the *PURO* cassette we could generate a chromosomal *BLAST/PURO RAD50*^*-/-*^ cell with no more intact *RAD50* chromosomal copy (Fig 1B, lane 4) but with the presence of the extrachromosomal rescue *RAD50* copy with its diagnostic 4.9 kb SacI-SacI band (Fig 1A) hybridizing with the *RAD50* ORF probe (Fig 1C, lane 4). Removing the drug NEO pressure (the marker of the rescuing episome) for several passages would lead to either maintenance or loss of the plasmid depending on whether *RAD50* is essential or not. Cells grown in absence of selection for the *NEO* marker maintained the episome (up to 55 passages) (Fig 1C, lane 5). This was not due to an unusual stability of the plasmid since introduction of the same *NEO* plasmid in WT cells and then growth in absence of selection pressure led to the loss of the rescuing episome after 35 passages (Fig 1C, lanes 2, 3).

To further investigate the essentiality of the *RAD50* gene, we generated a mutated version of the Psp-*NEO*-*RAD50* after introduction of the K42A mutation in the RAD50 ATPase domain (Psp-*NEO*-*RAD50*^*K42A*^) (S2A Fig). The wild-type and mutated recombinant proteins were purified and the K42A mutation indeed impaired the ATPase activity of RAD50 (S2D Fig). The Psp-*NEO*-*RAD50*^*K42A*^ construct was transfected in the *BLAST RAD50*^*-/+*^ cells. In contrast to cells complemented with Psp-*NEO*-*RAD50*^*WT*^ (Fig 1B, lane 4, S2B Fig, lane 4), cells complemented with Psp-*NEO*-*RAD50*^*K42A*^ showed a remaining *RAD50* allele after integration of the *PURO* marker (S2B Fig, lane 5). The presence of the Psp-*NEO*-*RAD50*^*K42A*^ plasmid was confirmed by hybridization with a *RAD50* probe (S2C Fig). This result indicates that the ATPase activity of the rescue RAD50 copy is necessary to allow inactivation of both *RAD50* chromosomal alleles.

While inactivation of *RAD50* was not possible in a WT background, it was easily achieved in a *MRE11*^*-/-*^ mutant. Indeed an *MRE11*^*-/-*^ mutant was already available [38] and its *RAD50* locus was shown here to be intact, as a 3.2 kb fragment was present after SacI digestion of the genomic DNA (Fig 1B and 1C, lane 6). In the *MRE11*^*-/-*^ background we could inactivate both *RAD50* alleles with the *BLAST* and *PURO* markers without the need for a rescuing plasmid (Fig 1B and 1C, lane 7). To confirm the absence of both *MRE11* and *RAD50* genes in the *MRE11*^*-/-*^*RAD50*^*-/-*^ strain, we performed PCR amplification using two sets of primers (Fig 1D). The use of primers sets aa’ and bb’ should only amplify a PCR fragment if the *MRE11* and *RAD50* genes are present respectively. As expected, no PCR amplification was detected for both *MRE11* and *RAD50* genes in the *MRE11*^*-/-*^*RAD50*^*-/-*^ strain (Fig 1D, lane 7). We also carried out qRT-PCR for *RAD50* mRNA levels in a number of lines and inactivation of one *RAD50* allele by either PURO or BLAST reduced the mRNA by half compared to WT (S1C Fig, lanes 2,3). A similar fold decrease was observed in the *PURO/BLAST RAD50*^*-/-*^ cells that have an extra copy of the gene (S1B Fig, lane 4), indicating that this new allele is actively expressed (S1C Fig, lane 4). The level of RAD50 mRNA in the *MRE11*^*-/-*^ cells was similar to the WT strain but was undetectable in the *MRE11*^*-/-*^*RAD50*^*-/-*^ cells (S1C Fig, lanes 5,6). Overall, a nice correlation between *RAD50* copy number and mRNA expression was observed.

We also attempted inactivating the *RAD50* gene in cells with only one *MRE11* allele but mutated for its nuclease activity (*HYG/PUR-MRE11*^*H210Y*^ [38]). We failed to generate a RAD50 null mutant in this *HYG/PUR-MRE11*^*H210Y*^ cells since we observed the maintenance of a third *RAD50* chromosomal allele after integration of both *BLAST* and *NEO* resistant markers in the *RAD50* locus (S3B Fig, lane 3). *RAD50* thus appears to be essential in MRE11^H210Y^ nuclease dead cells.

The availability of a *RAD50* null mutant (in the *MRE11*^*-/-*^ background) has allowed to test for a number of phenotypes. The *MRE11*^*-/-*^ and *MRE11*^*-/-*^*RAD50*^*-/-*^ mutants had similar growth properties (S4A Fig), susceptibility to the DSBs inducing alkylating damaging agent methyl methanesulphonate (MMS) (S4B Fig); and displayed a reduced ability to carry out homologous recombination (HR) (S4E Fig). The *RAD50*^*-/-*^ mutant with its episomal rescue had similar growth phenotype and recombination proficiency as the WT cells (S4 Fig). To ensure that the phenotypes observed in the *MRE11*^*-/-*^ null mutant were not due to a reduction in RAD50 protein levels, we overexpressed RAD50 as part of an episomal construct (Psp-*RAD50*) in the *MRE11*^*-/-*^ cells. The *MRE11*^*-/-*^ and *MRE11*^*-/-*^ Psp-*RAD50* cells had similar growth properties and susceptibility to MMS (S4C and S4D Fig).

### DNA amplification in *LiMRE11*^*-/-*^*RAD50*^*-/-*^ parasites selected for methotrexate resistance

**Fig 2:**
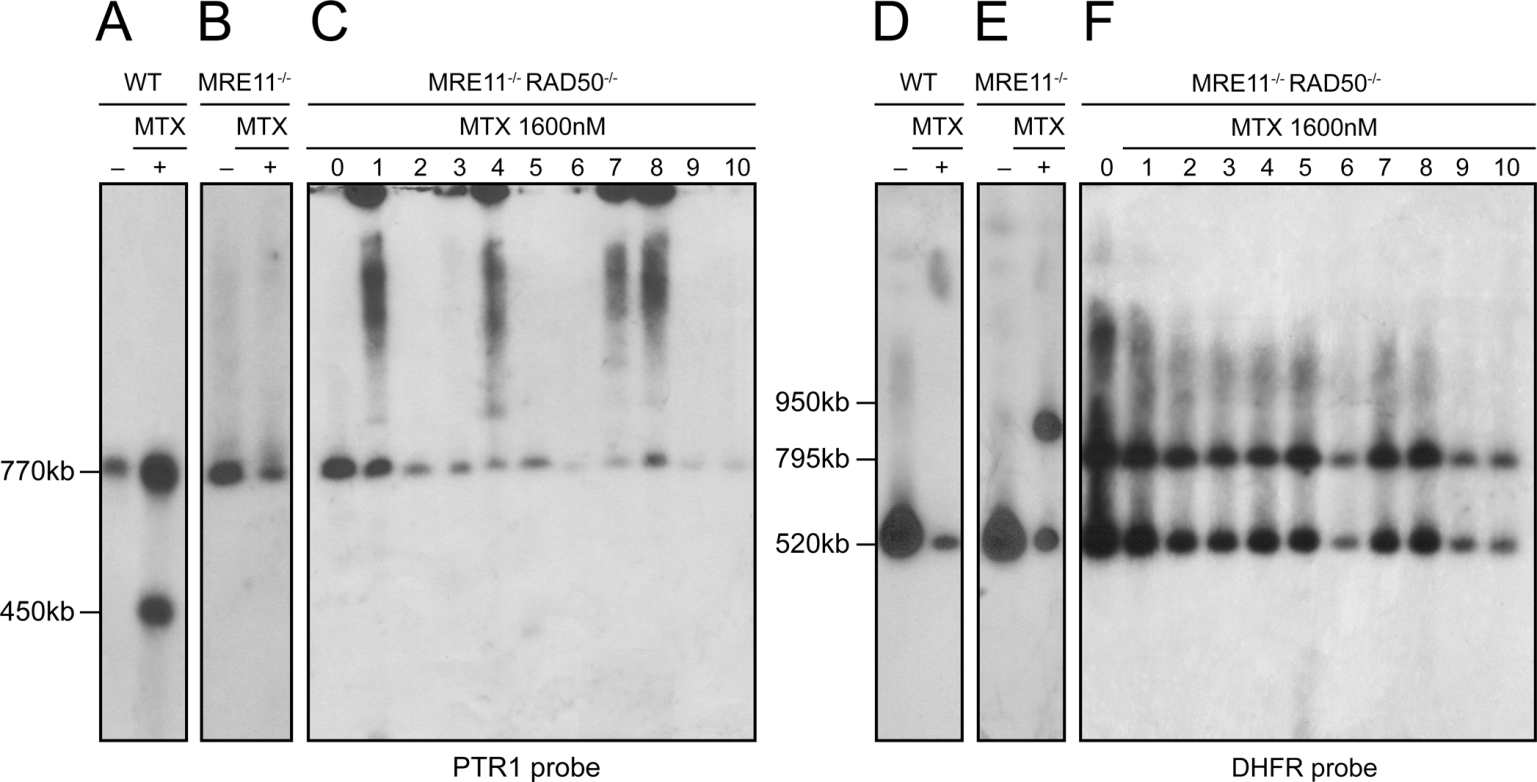
Gene amplification and rearrangement in *L*.*infantum MRE11*^*-/-*^*RAD50*^*-/-*^ cells selected for methotrexate (MTX) resistant cells. **(A, D)** *L*. *infantum* WT clone (lane +), **(B, E)** *L*. *infantum MRE11*^*-/-*^ clone (lane +) and **(C, F)** *L*. *infantum MRE11*^*-/-*^*RAD50*^*-/-*^ clones (lanes 1–10) were selected for MTX resistance up to 1600nM MTX, their chromosomes were separated by pulsed-field gel electrophoresis using a separation range between 150kb and 1500kb, transferred on membranes then hybridized with *PTR1* **(A, B, C)** and *DHFR-TS* **(D, E, F)** probes. Lanes 0 and lanes—are parasites without drug selection.

In response to drug pressure, *Leishmania* amplifies specific portion of its genome either as part of extrachromosomal circular or linear amplicons. Circles are dependent on RAD51 and RAD51-4 [14,48] while linear amplicons depend on MRE11 [38]. Selection of *Leishmania* WT cells for resistance to the antifolate methotrexate (MTX) often leads to the extrachromosomal amplification of the pteridine reductase gene *PTR1* (usually as part of linear amplicons) or of the dihydrofolate reductase-thymidylate synthase gene *DHFR-TS* (usually as part of circular amplicons) [49]. An example of a *PTR1* containing linear amplicon (at 450 kb) is provided in a WT cell that was selected fro MTX resistance (Fig 2A). The 770 kb *PTR1* hybridizing band corresponds to the chromosomal alleles. We showed previously that in contrast to WT cells, the *MRE11*^*-/-*^ mutant selected for MTX resistance did not have *PTR1* amplified as part of linear amplicons (Fig 2B) [38]. We investigated the ability of the *MRE11*^*-/-*^*RAD50*^*-/-*^ null mutants to perform extrachromosomal amplification by selecting clones for MTX resistance in a stepwise manner (up to 1600 nM, a 16-fold increase in resistance compared to parent cells). *Leishmania* chromosomes extracted from ten MTX resistant clones derived from *MRE11*^*-/-*^*RAD50*^*-/-*^ parasites were separated by pulse field gel electrophoresis (PFGE) (S5 Fig) and hybridized with the *PTR1* gene. Hybridization data revealed the 770 kb *PTR1* containing chromosome but no hybridizing bands diagnostic for *PTR1* linear amplicons (Fig 2C and S5 Fig). However, clones 1, 4, 7 and 8 had a *PTR1* circular amplification, as deduced from the characteristic hybridization profiles of circles in PFGEs, including the hybridization in the slots (corresponding to open circles) and the hybridizing smears (corresponding to topoisomers of the circles) [50] (Fig 2C lanes 1, 4, 7, 8). Amplification of the *DHFR-TS* gene is rarely observed in *L*. *infantum* selected for MTX resistance and this was further confirmed, where only the 520 kb chromosomal copies hybridized to a *DHFR* probe (Fig 2D). However since several resistant mutants had no *PTR1* amplification (Fig 2C) we hybridized the same resistant clones with a *DHFR-TS* probe. We observed the 520 kb band corresponding to the *DHFR-TS* containing chromosome but no sign for either circular or linear amplicons (Fig 2F). However, we detected in all clones a 795 kb band that surprisingly was also present in the parent *MRE11*^*-/-*^*RAD50*^*-/-*^ cells before MTX exposure (Fig 2F, lane 0). A similar, but clearly not identical (950 kb vs 795 kb) chromosomal rearrangement was previously observed in the *MRE11*^*-/-*^ strain after MTX pressure (Fig 2E, lane +) [38], suggesting that this locus is prone to chromosomal rearrangement. The chromosomal rearrangement involving the *DHFR-TS* chromosome in the *MRE11*^*-/-*^*RAD50*^*-/-*^ strain was further studied by genome sequencing of the nuclease deficient strains.

### Chromosomal translocations identified by next-generation sequencing analysis in the nuclease null mutants

**Fig 3:**
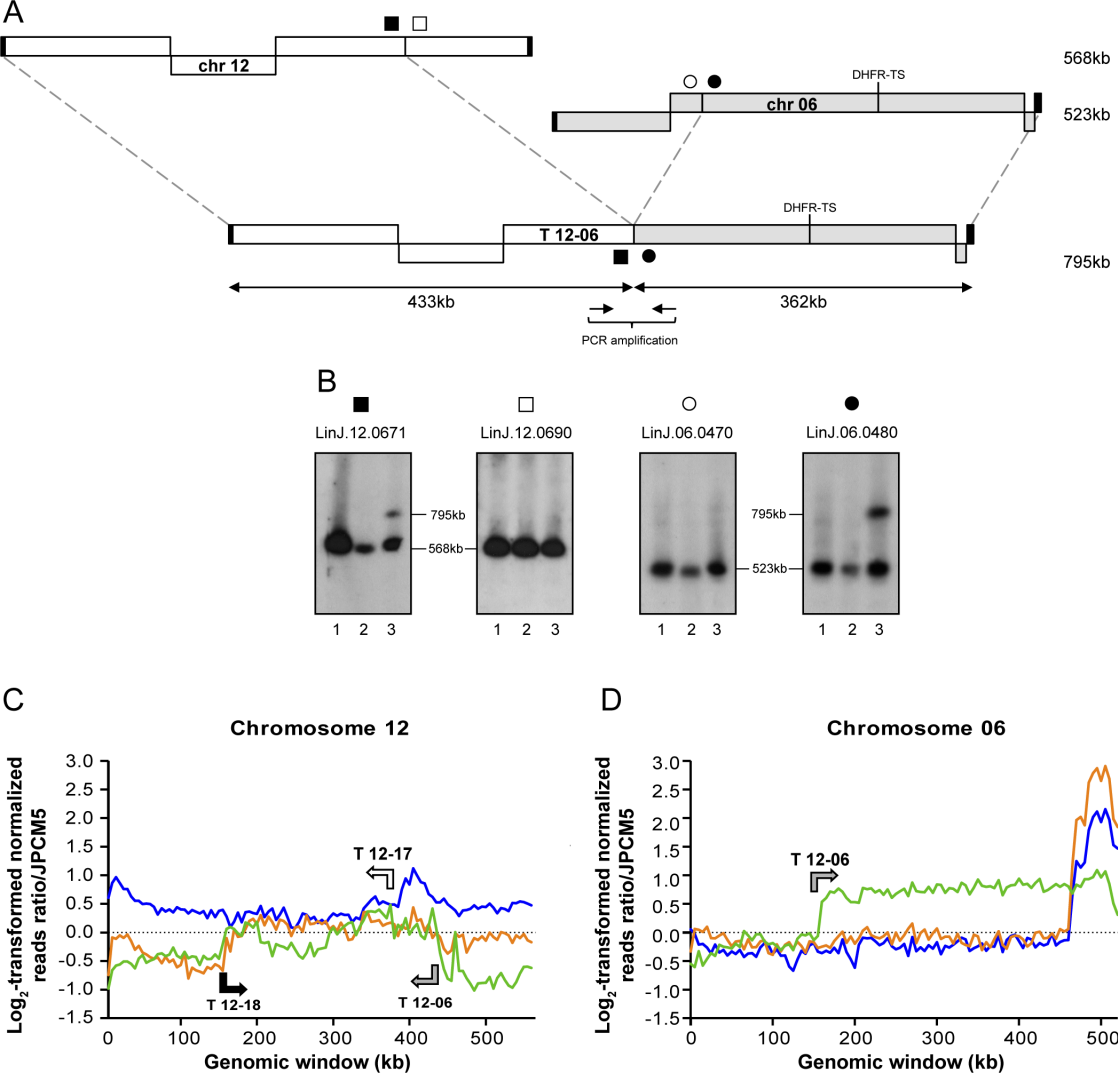
Translocation between chromosome 12 and 06 in *L*. *infantum MRE11*^*-/-*^*RAD50*^*-/-*^ cells. **(A)** Schematic representation of the translocation T 12–06 between chromosomes 12 and 06. **(B)** *L*. *infantum* chromosomes were separated by pulsed-field gel electrophoresis, transferred on membranes then hybridized with probes from LinJ.12.0671 (■), LinJ.12.0690 (□), LinJ.06.0470 (○) and LinJ.06.0480 (●). Lanes: 1, *L*. *infantum* WT; 2, *MRE11*^*-/-*^ and 3, *MRE11*^*-/-*^*RAD50*^*-/-*^. **(C, D)** Reads were mapped to the *L*. *infantum* JPCM5 genome and log_2_-transformed normalized read counts for non-overlapping 5 kb genomic windows are shown for chromosomes 12 and 06. Chromosome 12 is triploid in our *L*. *infantum* JPCM5 WT strain and a log_2_ fold change of 0,5 would suggest that chromosome 12 is tetraploid in *L*. *infantum* 263 WT. Arrows indicate direction and breakpoints of the translocations. Blue, *L*. *infantum* 263 WT; orange, *LiMRE11*^*-/-*^ and green, *LiMRE11*^*-/-*^*RAD50*^*-/-*^.

**Fig 4:**
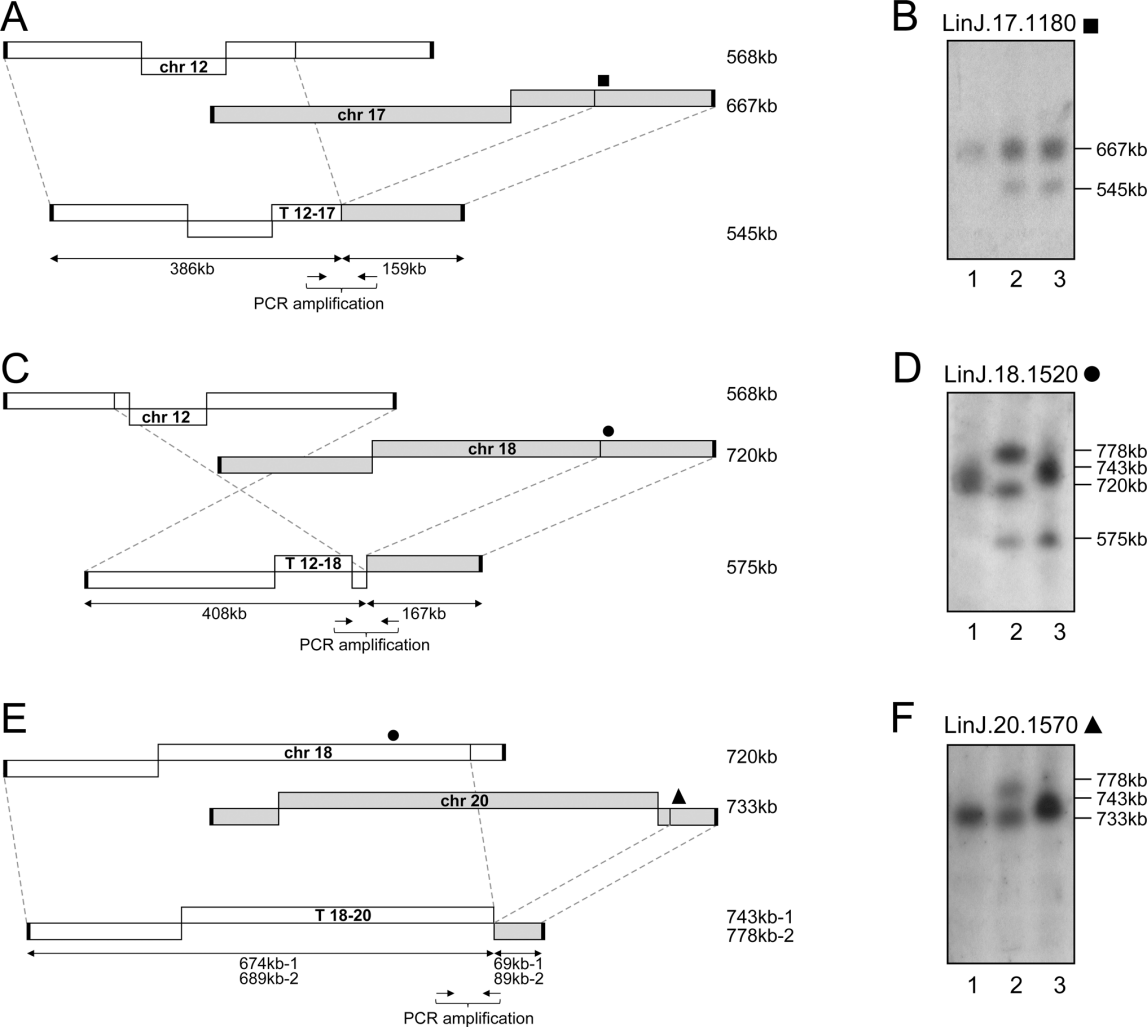
Translocation events in *L*. *infantum MRE11* and *RAD50* null mutants. **(A, C, E)** Schematic representation of the translocations T 12–17 **(A)**, T 12–18 **(C)** and T 18–20 **(E)**. **(B, D, F)** *L*. *infantum* chromosomes were separated by pulsed-field gel electrophoresis, transferred on membranes then hybridized with probes from LinJ.17.1180 (■), LinJ.18.1520 (●), LinJ.20.1570 (▲). Lanes: 1, *L*. *infantum* WT; 2, *MRE11*^*-/-*^ and 3, *MRE11*^*-/-*^*RAD50*^*-/-*^.

**Fig 5:**
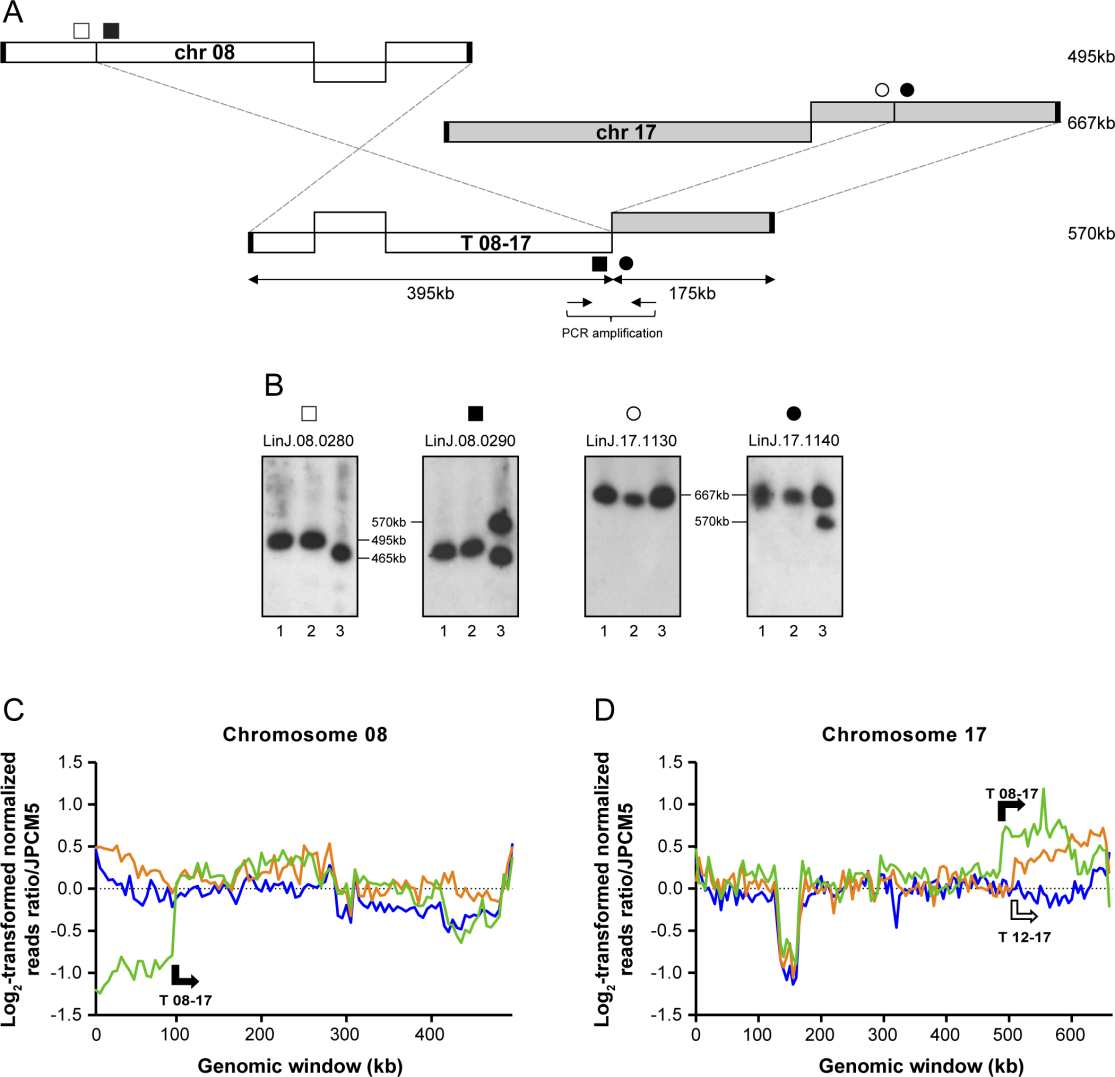
Translocation between chromosome 08 and 17 in *L*. *infantum MRE11*^*-/-*^*RAD50*^*-/-*^ cells. **(A)** Schematic representation of the translocation T 08–17 between chromosomes 08 and 17. **(B)** *L*. *infantum* chromosomes were separated by pulsed-field gel electrophoresis, transferred on membranes then hybridized with probes from LinJ.08.0280 (□), LinJ.08.0290 (■), LinJ.17.1130 (○) and LinJ.17.1140 (●). Lanes: 1, *L*. *infantum* WT; 2, *MRE11*^*-/-*^ and 3, *MRE11*^*-/-*^*RAD50*^*-/-*^. **(C, D)** Log_2_-transformed normalized read counts for non-overlapping 5 kb genomic windows on chromosomes 08 and 17. The Y-axis indicates log_2_ fold change from an initial diploid state for chromosomes 08 and 17. Arrows indicate direction and breakpoints of the translocations. Blue, *L*. *infantum* 263 WT; orange, *LiMRE11*^*-/-*^ and green, *LiMRE11*^*-/-*^*RAD50*^*-/-*^.

The genomes of the WT, *MRE11*^*-/-*^ and *MRE11*^*-/-*^*RAD50*^*-/-*^ lines were subjected to Illumina next-generation paired-ends sequencing (NGS). Sequencing reads were first aligned to the genome of *L*. *infantum* JPCM5 using bwa-mem alignment [51]. The alignments were then screened for discordant read pairs and split reads alignments using the Lumpy-sv and the Delly software [52,53]. This provided a list of chromosomal translocations present in the *MRE11* and *MRE11*/*RAD50* null mutants. A total of five translocations were observed in the MRE11 and MRE11/RAD50 deficient cells (Table 1). The analysis of the genomic sequences allowed the detection of the translocation of part of the *DHFR-TS* chromosome observed in Fig 2F. It involved 433 kb of chromosome 12 and 362 kb of chromosome 06 (encoding *DHFR-TS*), giving a hybrid chromosome T 12–06 of 795 kb (Fig 3A). To further characterize experimentally this translocation, we hybridized the chromosomes of the WT, *MRE11*^*-/-*^ and *MRE11*^*-/-*^*RAD50*^*-/-*^ cells with probes spanning the translocation breakpoints (filled and open squares and circles in Fig 3A). Hybridization with the gene *LinJ*.*12*.*0671* (■) revealed a band corresponding to chromosome 12 (568 kb) and an additional 795 kb band corresponding to the T 12–06 translocation in the *MRE11*^*-/-*^*RAD50*^*-/-*^ strain (Fig 3B). The same 795 kb band hybridized to *LinJ*.*06*.*0480* (●), a gene derived from chromosome 06 and part of the T 12–06 translocation (Fig 3B). The genes *LinJ*.*12*.*0690* (□) and *LinJ*.*06*.*0470* (○) should not be part of the hybrid chromosome (Fig 3A) and indeed when these genes were used as probes they only hybridized to bands corresponding to chromosomes 12 and 06 respectively (Fig 3B, Table 1). Two additional translocations also implicated chromosome 12 (T 12–17, T 12–18, Table 1) as described below. This is possible because several chromosomes of *Leishmania* are polyploids [13,54–56] and read counts indicate that chromosome 12 is tetraploid in our *L*. *infantum* WT strain (Fig 3C). One translocation led to a hybrid composed of 386 kb of chromosome 12 fused to 159 kb of chromosome 17 (Fig 4A). The size of chromosome 12 and the hybrid chromosome are too similar for their discrimination by PFGE but hybridization with *LinJ*.*17*.*1180* (■) showed a band corresponding to chromosome 17 (667 kb) and the 545 kb band corresponding to T 12–17 (Fig 4B). The third translocation implicating chromosome 12 involved chromosome 18 (408 kb of chromosome 12 and 167 kb of chromosome 18 leading to an hybrid chromosome of 575 kb, (Fig 4C)). The size of chromosome 12 and the hybrid were again too similar for discrimination by PFGE but hybridization with *LinJ*.*18*.*1520* (●) revealed the 575 kb band corresponding to T 12–18 (Fig 4D lanes 2, 3). The hybridization patterns in the two nuclease mutants are more complex with no band exactly migrating with the intact chromosome 18 band (Fig 4D). In the case of the *MRE11*^*-/-*^*RAD50*^*-/-*^ mutant, this can be explained in part by an additional translocation of chromosome 18 with chromosome 20 (Fig 4E) where the new hybrid chromosome (T 18–20) had 674 kb of chromosome 18 and 69 kb of chromosome 20 for an estimated length of 743 kb (Fig 4D and 4F lane 3). In the *MRE11*^*-/-*^ mutant we observed three bands hybridizing with the *LinJ*.*18*.*1520* (●) probe (Fig 4D, lane 2). The band of 575 kb corresponds to the T 12–18 hybrid chromosome, the highest band at 778 kb corresponds to one of two versions of T 18–20 (with an internal duplication that was highlighted by reads depth analysis, see below). This band also hybridized with *LinJ*.*20*.*1570* (▲) (Fig 4F, lane 2). The middle band hybridizing to *LinJ*.*18*.*1520* (●) appears slightly smaller than the 720 kb WT chromosomal copy (Fig 4D, lanes 1 and 2) and may correspond to a truncated form of chromosome 18. The final translocation highlighted by NGS involved chromosome 08 and 17 (Table 1) and T 08–17 consists of 175 kb of chromosome 17 and 395 kb of chromosome 08 leading to an hybrid chromosome of 570 kb (Fig 5A). Hybridization with *LinJ*.*08*.*0290* (■) revealed a 570 kb band corresponding to T 08–17 and a 465 kb band slightly smaller than the expected size of chromosome 08 (495 kb) (Fig 5B, lane 3). This smaller 465 kb band also hybridized with *LinJ*.*08*.*0280* (□) (not part of T 08–17), and may, similarly to one copy of chromosome 18 discussed above, correspond to an internal deletion or truncation of the original chromosome but the bioinformatics analysis did not provide support for these potential scenarios. Hybridization with probes derived from chromosome 17 further supported the formation of the hybrid chromosome T08-17 (Fig 5B, lane 3).

**Table 1:** Translocation breakpoints mapping and genomic features at the breakpoints.

chromosome	length	breakpoint position	translocated genes	microhomology	translocation found	
	(kb)				*MRE11*^*-/-*^	*MRE11*^*-/-*^*RAD50*^*-/-*^
12	568	432550—ORF LinJ.12.0671	LinJ.12.0001—LinJ.12.0671	9 bp	* *	* *
06	523	160868—ORF LinJ.06.0480	LinJ.06.0480—LinJ.06.1360			
T 12–06	795				* *	*√*
12	568	385543—intergenic region	LinJ.12.0001—LinJ.12.0680	not determined	* *	* *
17	667	507736—intergenic region	LinJ.17.1170—LinJ.17.1590		* *	* *
T 12–17	545				*√*	* *
12	568	159609—intergenic region	LinJ.12.0310—LinJ.12.0930	17 bp	* *	* *
18	720	552566—intergenic region	LinJ.18.1330—LinJ.18.1670		* *	* *
T 12–18	575				*√*	*√*
18	720	673844—ORF LinJ.18.1530	LinJ.18.0010—LinJ.18.1530	7 bp	* *	* *
20	733	664091—ORF LinJ.20.1560	LinJ.20.1560—LinJ.20.1790		* *	* *
T 18–20	743				*√*	*√*
08	495	100048—intergenic region	LinJ.08.0290—LinJ.08.1310	11 bp	* *	* *
17	667	492048—intergenic region	LinJ.17.1140—LinJ.17.1590			
T 08–17	570				* *	*√*

The Lumpy-sv and Delly software also revealed a fusion between chromosome 27 and chromosome 02 that was already present in the WT cells, highlighting a difference between the *L*. *infantum* 263 WT strain compared to the reference *L*. *infantum* JPCM5 WT (S6 Fig). Most of chromosome 27 (1044 kb) is fused with the last two genes on chromosome 02 (4 kb) (S6A Fig). Sequence homology between the end of chromosomes 2 and 27 has already been described for *L*. *major* [57] with subtelomeric repeats and this rearrangement occurring in the WT may correspond to telomere exchange rather than translocation.

### Copy number variations in the *MRE11* and *MRE11*/*RAD50* null mutants

In the past, we have used normalized reads depth coverage over the 36 chromosomes to predict copy number variations [11,12]. Sequenced reads of the 36 chromosomes of the *L*. *infantum* 263 strain indicated that while the majority of chromosomes were mostly diploid, chromosomes 12, 13 and 31 were polyploid. There were no changes in ploidy in the nuclease mutants except when translocation occurred. Normalized log_2_-transformed read counts for chromosome 06 in WT cells, *MRE11*^*-/-*^, and *MRE11*^*-/-*^*RAD50*^*-/-*^ revealed a shift at the T 12–06 breakpoint in the *MRE11*^*-/-*^*RAD50*^*-/-*^ null mutant, leading to an increased number of reads for part of chromosome 6 starting with gene *LinJ*.*06*.*0480* (Fig 3D). At one telomere end of chromosome 06 in WT and *MRE11*^*-/-*^ strains we observed increased number of reads for a region of 60 kb (Fig 3D) that corresponds to a linear extrachromosomal amplicon that we have previously characterized in *L*. *infantum* 263 WT [14]. This linear amplicon was lost in the *MRE11*^*-/-*^*RAD50*^*-/-*^ strain. Reads depth coverage indicated that chromosome 12 is tetraploid in *L*. *infantum* 263 WT and that *MRE11*^*-/-*^ and *MRE11*^*-/-*^*RAD50*^*-/-*^ parasites probably contained only one intact copy of chromosome 12 (Fig 3C) but all the hybrid chromosomes T 12–06, T 12–17 and T 12–18 contribute to a higher ploidy for most sequences of chromosome 12 (Fig 3C). The normalized reads depth of chromosome 12 also highlighted the T 12–06, T 12–17 and T 12–18 breakpoints (Fig 3C). Similarly, the breakpoints for T 12–18 and T 18–20 also fitted with a change in reads depth on chromosome 18 and chromosome 20 (S7 Fig). In the case of chromosome 18, reads depth showed a shift at the T 12–18 and T 18–20 breakpoints in the two mutants and overlapping regions between T 12–18 and T 18–20 (from *LinJ*.*18*.*1330* to *LinJ*.*18*.*1530*) were present in three copies (S7A Fig). In the *MRE11*^*-/-*^ strain, normalized read counts for chromosome 18 also highlighted internal duplication of 15 kb close to the T 18–20 breakpoint, increasing the size of the T 18–20 (S7A Fig). In the same translocation, part of chromosome 20 also showed a duplication of 20 kb (S7B Fig) in the *MRE11*^*-/-*^ cells, increasing the size of the translocation T 18–20 from 743 kb to 778 kb in that strain (Fig 4E). Reads mapping to chromosome 20 also showed a decreased number of reads at one telomere end in the *MRE11*^*-/-*^*RAD50*^*-/-*^ cells (S7B Fig), a phenomenon that we observed for several other chromosomes (see below). Finally, normalized reads depth coverage over chromosomes 08 and 17 highlighted the T 08–17, T 12–17 and T 08–17 breakpoints (Fig 5C and 5D).

### Determination of translocation breakpoints by PCR

**Fig 6:**
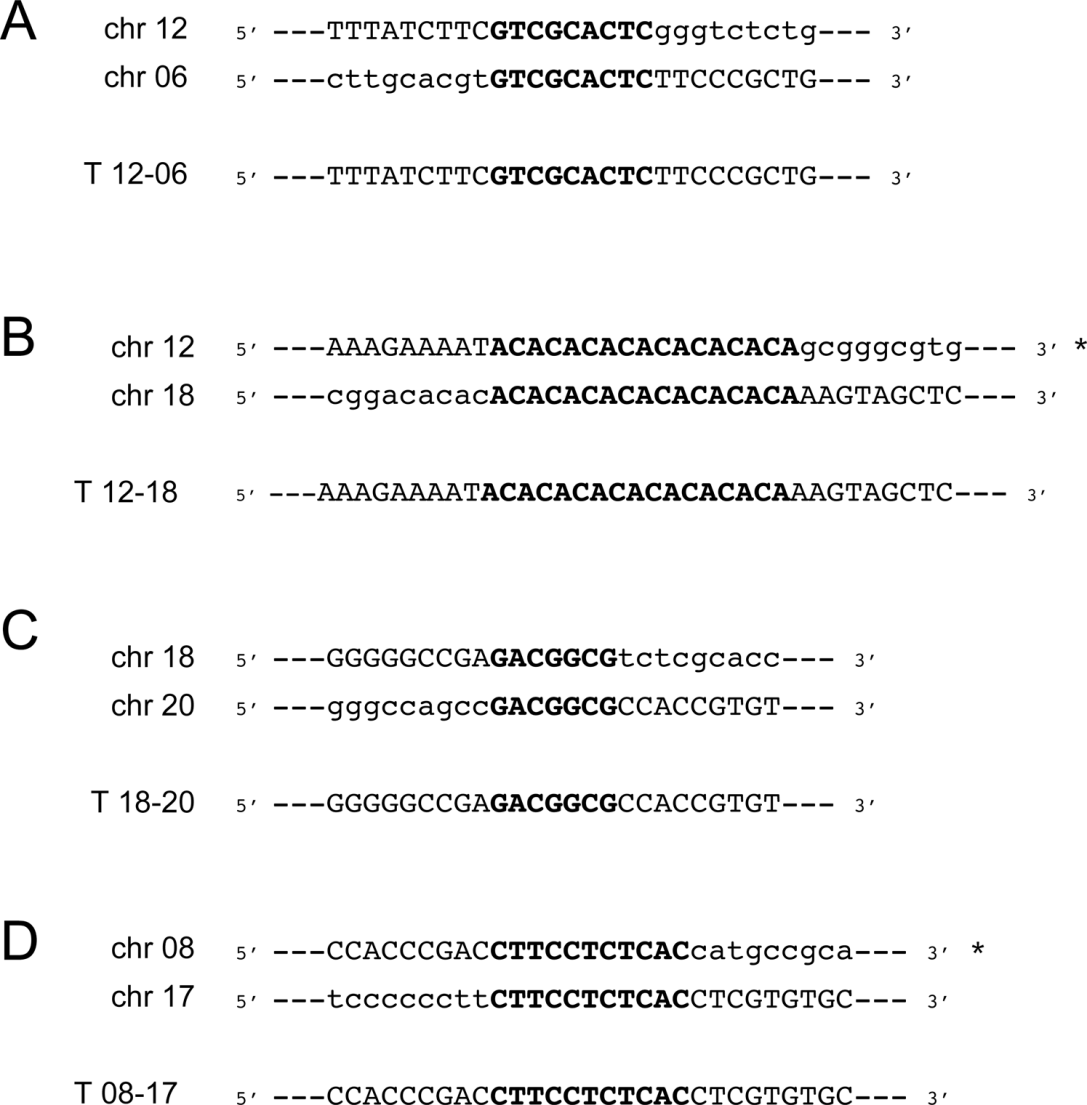
Sequence microhomology at translocation breakpoints. DNA sequences obtained from direct sequencing of the junctions T 12–06 **(A)**, T 12–18 **(B)**, T 18–20 **(C)** and T 08–17 **(D)**. Sequences of wild-type parasites found close to breakpoints were aligned to the respective chromosomes involved as well as the resulting hybrid chromosome. Microhomology sequences are highlighted in capital bold letters. Asterisk indicates that the sequence corresponds to the antisense strand.

We used PCR to validate the new junctions created by the fusion of portions of chromosomes in all translocations. Oligonucleotide primers located on each side of the translocation points were designed to target the new junctions. The ORFs identities (or position of intergenic regions) closest to the breakpoints can be found in Table 1. We were able to precisely define the junction of 4 of the 5 translocations (T 12–06, T 12–18, T 18–20 and T 08–17). In all cases the sequencing of the fusion points in the hybrid chromosomes revealed that the rearrangements occurred at the level of microhomologies between 7 and 17 bp (Fig 6A–6D). There were no common sequence features between the various repeats. We were unsuccessful to map precisely the translocation breakpoints for T 12–17. Indeed the breakpoint is located inside a region containing repeated DNA sequences along 60 kb and specific PCR amplification of the junction has not been possible. The genome of *Leishmania* is constituted of large polycistronic clusters of genes that are co-expressed [58,59]. Interestingly some of the translocation would create new regions where co-directional gene clusters diverge or converge (T 12–18, T 18–20, T 08–17) that may impact on gene expression.

PCR amplification of the junction between chromosomes 27 and 02 revealed an insertion of 21 bp at the junction of chromosome 27 and chromosome 02 in the 27–02 hybrid (S6B Fig). This rearrangement is clearly not similar to the translocation events characterized in this study and may correspond to exchange of telomeric sequences between chromosomes 27 and 02.

### Decrease of read counts at subtelomeric loci in the absence of RAD50

**Fig 7:**
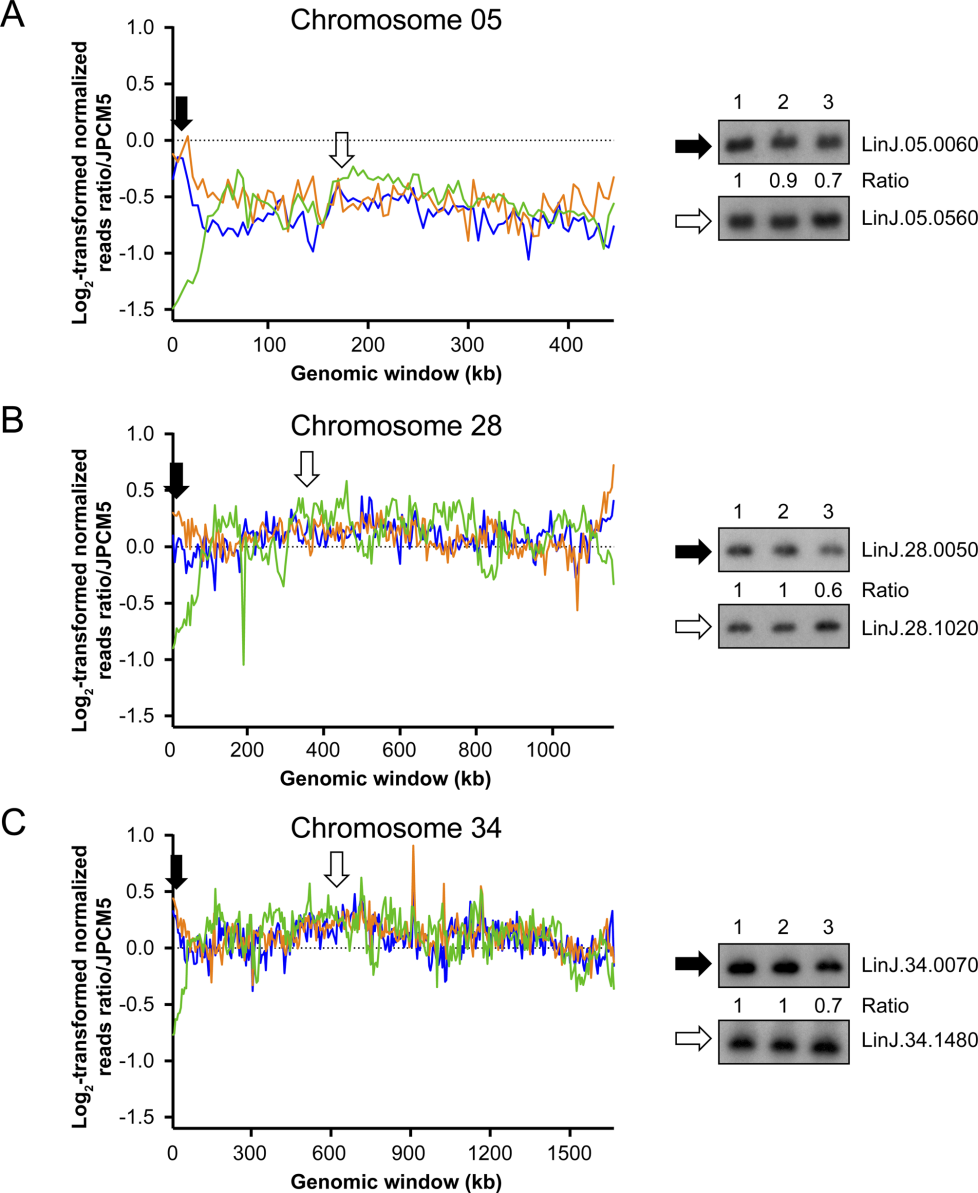
Reduction of mapped read counts at subtelomeric locations in *L*. *infantum MRE11*^*-/-*^*RAD50*^*-/-*^ cells. Log_2_-transformed normalized read counts on chromosome 05 **(A)**, chromosome 28 **(B)** and chromosome 34 **(C)**. Blue, *L*. *infantum* 263 WT; orange, *LiMRE11*^*-/-*^ and green, *LiMRE11*^*-/-*^*RAD50*^*-/-*^. Black arrows define the location of probes derived from genes *LinJ*.*05*.*0060*, *LinJ*.*28*.*0050* and *LinJ*.*34*.*0070*. White arrows define the location of probes derived from control genes *LinJ*.*05*.*0560*, *LinJ*.*28*.*1020* and *LinJ*.*34*.*1480* that were used for Southern blot hybridization of PvuII-digested genomic DNAs. Lanes: 1, *L*. *infantum* WT; 2, *LiMRE11*^*-/-*^ and 3, *LiMRE11*^*-/-*^*RAD50*^*-/-*^.

The *MRE11*^*-/-*^*RAD50*^*-/-*^ parasites displayed a decreased number of reads mapping to chromosomes ends, suggesting sequences near telomeres were impaired in that strain. This phenomenon occurred for eleven chromosomes (Fig 7 and S8 Fig) and three were experimentally verified by Southern blot (Fig 7). Genomic DNAs from the WT, *MRE11*^*-/-*^ and *MRE11*^*-/-*^*RAD50*^*-/-*^ cells were hybridized with a chromosome 05 probe close to the telomeres (*LinJ*.*05*.*0060*) and hybridization intensities were compared with probe *LinJ*.*05*.*0560* used as an internal control for DNA loading (Fig 7A). Hybridization intensities yielded a 0.7 fold-decrease for the *MRE11*^*-/-*^*RAD50*^*-/-*^ strain compared to the WT or *MRE11*^*-/-*^ cells. Similar analyses were performed with probes derived from chromosome 28 (Fig 7B) and chromosome 34 (Fig 7C) and when telomeric proximal probes were used the signal was consistently lower in the *MRE11*^*-/-*^*RAD50*^*-/-*^ parasites, compared to either WT cells or the *MRE11*^*-/-*^ mutant. Genomic DNAs from WT, *MRE11*^*-/-*^ and *MRE11*^*-/-*^*RAD50*^*-/-*^ cells were also digested with Sau3aI, AluI and RsaI and hybridized with a telomeric probe [60,61]. After hybridization, discrete bands were present in both the WT and *MRE11*^*-/-*^ but in the *MRE11*^*-/-*^*RAD50*^*-/-*^ cells we observed a smear (S9A Fig). When an internal probe far from telomere was used (*PTR1* gene), a single band was observed in all three lines (S9B Fig). The results suggest that within the *MRE11*^*-/-*^*RAD50*^*-/-*^ population there is considerable heterogeneity at the end of chromosomes in individual cells (explaining the smear when hybridized with a telomeric probe), in line with the decreased number of reads mapping chromosomes ends (Fig 7).

## Discussion

Gene rearrangement in *Leishmania* is genome wide [14,38] and can lead to extrachromosomal elements [13,55], to chromosomes in multiple copies and to mosaic aneuploidy [56]. It is thought that these events can lead to selective advantage [11–14] and our recent work has shed some light on the enzymes involved in these processes. RAD51 and at least one of its paralog (RAD51-4) are involved in the formation of extrachromosomal circles [14,48], while MRE11 is involved in the formation of linear amplicons [38]. MRE11 is partnering with RAD50 and NBS1 as part of the MRN complex [34,35]. MRE11 is one of the main sensor of DNA DSBs while RAD50 modulate the activity of the complex [8]. It was shown in *Saccharomyces cerevisiae* that the RAD50 coiled-coil domain is indispensable for MRE11 functions since truncation of this domain in RAD50 impaired telomere maintenance, meiotic DSB formation, HR and NHEJ, indicating its need for MRN activities [62]. We initiated this work to test whether MRE11 and RAD50 functions would overlap and whether these proteins are involved in the maintenance of genomic integrity. To test this we used gene inactivation and while we were able to obtain a *MRE11*^*-/-*^ null mutant [38], it has been impossible to generate a *RAD50*^*-/-*^ null mutant in a WT background (Fig 1 and S1 Fig). We could only inactivate both alleles if a rescue episomal copy of *RAD50* was present but despite prolonged passages in absence of the selecting drug, we could not lose the episomal *RAD50* copies, a strong suggestion that *RAD50* is essential in *Leishmania*. A chromosomal copy of *RAD50* was maintained upon gene inactivation if cells were complemented with a mutated *RAD50*^*K42A*^ rescue plasmid indicating that a fully functional RAD50 is essential for cell survival (S2 Fig). In mammals, RAD50 and MRE11 are essential [41,42] but in yeast both proteins are dispensable [33,63,64], thus diverse organisms have different requirements for proteins part of the MRN complex. In both human cells and *S*.*cerevisiae*, introduction of mutations in the *RAD50* ATPase domain impaired DNA binding and DNA unwinding [65] suggesting RAD50 is required for the stability of the DNA-MRN complex interaction [66,67]. We also tried to inactivate the *RAD50* gene in MRE11^H210Y^ nuclease-deficient cells but this did not lead to a *RAD50* null mutant (S3 Fig). The MRE11^H210Y^ protein is deficient in nuclease activity but still capable of DNA binding [38]. Our results provide good evidence that inactivation of *RAD50* may only be possible in the absence of *MRE11*. There may possibly be a need for RAD50 when MRE11 is present, even if its nuclease domain is inactivated such as in MRE11^H210Y^. The inactivation of MRE11 nuclease activity in murine cells did not change the MRN complex formation [36], and possibly the presence of MRE11 forces the presence of RAD50 and the formation of MRE11 ^H210Y^/RAD50 interactions. We infer that, in addition to its interaction with MRE11, the *Leishmania* RAD50 protein might also have important functions which would only occur in the presence of MRE11. Indeed inactivation of *RAD50* was easily achieved in a *MRE11*^*-/-*^ background (Fig 1B and 1C lanes 7). One hypothesis is that *MRE11* inactivation leads to genetic compensation in *Leishmania* and this compensation makes RAD50 dispensable to any other putative important function that RAD50 may have. It is also possible that MRE11 is detrimental in the absence of a RAD50-mediated regulation that might happened through maintenance of the MRE11/RAD50 complex stoichiometry [68]. This hypothesis was plausible with previous observations showing that overexpression of MRE11 in *Leishmania* was detrimental for cell growth [38], possibly because of stoichiometry disruption.

Absence of the MRE11/RAD50 complex led to a growth defect, a sensitivity to MMS and an altered capacity for HR (S4A and S4B Fig). Although knockdown of individual components of MRN in human cells led to a decrease in the other two MRN members [69], our results suggest that RAD50 is normally expressed in the *MRE11*^*-/-*^ strain at the RNA levels (S1C Fig) and functionally (S4C and S4D Fig). The MRE11 and RAD50 deficient cells had an incapacity of generating *PTR1* linear amplicons upon MTX selection (Fig 2 and [38]). Whole genome sequencing indicated that translocations were observed in mutants lacking a fully functional MRE11/RAD50 complex (Table 1) and the only clear difference between the *MRE11*^*-/-*^ and *MRE11*^*-/-*^*RAD50*^*-/-*^ mutants was at the level of subtelomeric sequences where the number of sequenced reads was much lower in several subtelomeric loci for the *MRE11*^*-/-*^*RAD50*^*-/-*^ mutant (Fig 7 and S8 Fig).

One new aspect of this work is the discovery of chromosomal translocations which have not been observed before in old world *Leishmania* species [70–72]. Studies in yeast have also indicated an increase of translocations events and chromosomal rearrangements when either *MRE11* or *RAD50* are mutated [2]. Translocations are likely to have occurred after DSBs which are usually repaired by either HR or NHEJ. Several components of NHEJ are absent in *Leishmania* and the parasite thus relies mostly on HR [23]. Since HR is diminished in the MRE11 and RAD50 mutants (S4E Fig), the cells may use alternative strategies to repair DNA. One of these alternative pathways used for repair of DSBs is based on MMEJ. Indeed, MMEJ has been implicated in chromosomal translocation in yeast [73], mammals [74], and in the related parasite *T*. *brucei* [75]. Mice defective in NHEJ have exhibited an increased level of translocations mediated by an alternative NHEJ that relied on microhomology [76]. The PCR reactions of the junctions created following translocations revealed that these events occurred via a mechanism of MMEJ where the microhomology is between 7 and 17 bp (Fig 6 and Table 1). There is no sequence specificity between the various translocation breakpoint sequences. We have shown previously that the *Leishmania* genome is filled with large repeated sequences [14] and we found that several of the microhomology sequences are either part of large repeated sequences (for T 12–17) or close to repeated sequences (for T 12–06 and T 12–18). It is well known that repeated sequences can be fragile sites and therefore more prone to DSBs and could explain translocation mediated by MMEJ [77–79]. A recent study done in *Leishmania donovani* has described the use of MMEJ for repair of Cas9-induced DSBs using the CRISPR-Cas9 system, even though the parasites mostly relied on HR for DSBs repair [80]. The Cas9/gRNA complex is, however, continuously generating DNA breaks in every chromosomal allele of the targeted region, complicating the search for intact homology by the HR machinery, hence favoring alternative end-joining mechanism such as MMEJ. When a template with sequence homology was provided to the parasites, the HR mechanism largely dominated the DSBs repair [80]. In our study, the homologous chromosomal allele is thought to be intact but the defect in HR probably led the cells to a MMEJ mechanism for DNA repair. Several studies done in yeast have shown the importance of resection by MRE11 for the first step of MMEJ [30,69,81–83] but we suggest MMEJ can be MRE11-independent in *Leishmania*. It is possible that upon genetic compensation in the knock-out strains, the expression of other nucleases is increased and could perform some of the activities usually carried out by MRE11. However, the other nucleases encoded by *Leishmania* (*e*.*g* EXO1, DNA2 [23]) are reputed for extensive DNA resection which is unfavorable for MMEJ that usually favors short length resections (performed by MRE11) [83,84]. Further experiments could help in deciphering the MRE11-independent MMEJ in *Leishmania*. Few chromosomes have been implicated in translocation and some of these chromosomes (*e*.*g* chromosomes 12, 17 and 18) have been implicated in more than one translocation. It is not clear whether the initial state of ploidy has a role to play seeing as chromosome 12 is tetraploid, chromosomes 17 and 18 are diploid but other chromosomes not involved in translocation are also polyploid like chromosomes 13, 31 and 32 (in *L*. *infantum* 263 WT). The rearrangements observed were also stable since re-sequencing of 3 clones from each null mutants after six months of continuous growth highlighted the same translocations and no additional one. Thus either *Leishmania* can support only few translocations or those are relatively rare events that can be maintained. Translocations and the formation of hybrid chromosomes change ploidy of specific regions (Figs 3C, 3D, 5C and 5D) but in general, a minimum of diploid state is conserved because of the overlap with the different translocations. Overall, none of the translocation breakpoints correspond to transcription initiation or termination sites and one aspect that was not studied is whether the formation of hybrids has consequences on the expression of genes in this novel context. This is particularly relevant as some of the rearrangements created regions where co-directional gene clusters diverge or converge (*e*.*g* T 12–18, T 18–20, T 08–17). Those divergent and convergent regions could represent regions where RNA polymerase can enter or exit, but transcription could also initiate or terminate within directional gene clusters [59,85,86]. Furthermore, when we compared our results to a recent study revealing that *Leishmania* chromosomes are replicated by a single origin (instead of multiple sites of replication origins as other eukaryotes) [87], we observed that the translocation events generated hybrid chromosomes that also contained a single origin of replication (coming from either one of the chromosome involved in the translocation). This observation suggest that even though genomic integrity was altered by the formation of hybrid chromosomes, the parasites were consistent in maintaining a single-origin of replication per chromosome.

In the *MRE11*^*-/-*^*RAD50*^*-/-*^ mutant, chromosome 8 is smaller than in the WT cells and in the *MRE11*^*-/-*^parasites, chromosome 18 is also smaller than in the WT, suggesting that some rearrangements events (*e*.*g* deletions) happened in the mutants. While analysis of sequence reads did not allow us to confirm this deletion on chromosome 8, sequence reads has been highly useful in the past to detect changes in copy number [11,88]. We nonetheless conducted a reads depth analysis to detect either deletions or duplications and the bioinformatics analysis revealed the potential presence of several of them in the null mutants. However experimental validation by Southern blot only allowed confirming 1 out of 5 deletions and 1 out of 5 duplications deduced from the bioinformatics analyses. This suggests that the 5 kb window given by our bioinformatics pipeline might not be optimal for the detection of deletion and duplication events. Nevertheless our results suggest that there are more than translocation events as part of gene rearrangements in the nuclease null mutants.

One key change that reads depth analysis detected and that we could confirm experimentally is a reduction of reads of several subtelomeric sequences exclusively in the *MRE11*^*-/-*^*RAD50*^*-/-*^ mutant (Fig 7 and S8 Fig), suggesting that the absence of RAD50 altered chromosome end stability as already observed in human cells [89]. The log_2_-transformed read counts would suggest that populations are not clonal but that several cells within the population have various levels of subtelomeres shortening including coding sequences (Fig 7). Decrease of sequence reads extends up to 100 kb from the telomeres in some of the cells although in general, the shortenings are smaller. The *T*. *brucei* subtelomeres harbor fragile sites [90] and subtelomeric regions are known to be more sensitive to DSBs that are processed differently than internal DSBs [91,92]. The decreased in sequence reads observed here is possibly due to an altered repair of DSBs in the *MRE11*^*-/-*^*RAD50*^*-/-*^ mutant that lead to shortening of subtelomeric sequences. Indeed, a Southern blot of DNAs derived from *MRE11*^*-/-*^*RAD50*^*-/-*^ hybridized with a telomeric probe revealed and hybridization smear suggesting considerable heterogeneity at chromosomes ends for individual cells within the population. RAD50 seems to have an important role in this since this phenomenon is not observed in the *MRE11*^*-/-*^ mutant.

This study provides strong evidence that *MRE11* gene knock-out is a prerequisite for *RAD50* inactivation in *Leishmania*. Chromosomal translocations are observed in the cells lacking a fully functional MRE11/RAD50 complex, and subtelomeric regions stability is altered in the absence of *RAD50*. Moreover, we report for the first time in *Leishmania* a MRE11-independent alternative end-joining mechanism that relies on microhomology sequences. Overall, these results show a predominant role of the two DNA repair proteins MRE11 and RAD50 in chromosomal organization. Deciphering DNA repair mechanisms and maintenance of genomic integrity in *Leishmania* parasites may allow novel strategies for their control as they seem to rely on gene amplification and rearrangement for surviving the changing environment in which they grow.

## Material and Methods

### Strains, culture conditions

Promastigotes of *Leishmania infantum* (MHOM/MA/67/ITMAP-263) and all recombinants were grown in SDM-79 medium at 25°C supplemented with 10% fetal bovine serum, 5μg/ml of hemin at pH7.0. Independent clones generated in this study were selected for methotrexate (MTX) resistance in M199 medium, using a stepwise selection starting from an EC_50_ of 100nM up to 1600nM of MTX. All chemical reagents were purchased from Sigma-Aldrich unless specified.

### Generation of *LiRAD50* (*LinJ*.*28*.*0560*) null mutant cells

The *L*. *infantum RAD50* null mutant (*RAD50*^*-/-*^) was obtained by targeted gene replacement. *RAD50* flanking regions were amplified from *L*. *infantum* 263 wild-type (WT) genomic DNA and fused to blasticidin-S deaminase (*BLAST*), puromycin acetyltransferase (*PURO*) and neomycin phosphotransferase (*NEO*) genes using a PCR fusion based-method as described previously [93]. Briefly, 5’UTR of *RAD50* was amplified using primers C and D for the *BLAST* cassette, primers C and E for the *PURO* cassette and primers C and F for the *NEO* cassette. The *BLAST*, *PURO* and *NEO* genes were amplified with primers G and H, I and J and K and L respectively. 3’UTR of *RAD50* was amplified using primers M and N for all inactivation cassettes (see primer sequences in S1 Table). At least 3μg of the *5’UTR-BLAST-3’UTR*, *5’UTR-PURO-3’UTR* or *5’UTR-NEO-3’UTR* linear fragments were transfected by electroporation (as described in [94]) in *L*. *infantum* WT, *L*. *infantum MRE11*^*-/-*^ or *L*. *infantum HYG/PUR-MRE11*^*H210Y*^ cells [38] to replace both *RAD50* alleles. Recombinants were selected in the presence of 80μg/ml of blasticidin-S hydrochloride, 100μg/ml of puromycin dihydrochloride (Wisent) and 40μg/ml G418 (Geneticin; Sigma-Aldrich). After 4–5 passages, cells resistant to the drug selection were cloned in SDM-Agar plates (1%) in the presence of the same concentrations of drugs. PCR analysis of the recombinants was done using forward primer located in the *MRE11* 5’ flanking region with reverse primer inside the *MRE11* gene (primers set aa’), and forward primer in the *RAD50* 5’ flanking region with reverse primers located inside the *RAD50* gene (primers set bb’) (see primers sequences in S1 Table).

### Episomal overexpression of RAD50

An episomal construct, Psp72-α-*NEO*-α-*RAD50*^*WT*^ was designed to express RAD50 in the cells before inactivation of the second *RAD50* genomic allele. Briefly, the *RAD50* gene was amplified by PCR using primers O and P from *L*. *infantum* WT genomic DNA. The amplified product was first cloned in pGEM-T_Easy_ vector and then subcloned in Psp72-α-*NEO*-α [95] in the HindIII and NdeI sites of the vector. Site-directed mutagenesis (Stratagene, Quickchange) was used to introduce the K42A mutation in the *RAD50* ORF and generate the Psp72-α-*NEO*-α-*RAD50*^*K42A*^ using primers Q and R (S1 Table). Both Psp72-α-*NEO*-α-*RAD50*^*WT*^ and Psp72-α-*NEO*-α-*RAD50*^*K42A*^ plasmids were then transfected by electroporation in the *L*. *infantum BLAST RAD50*^*-/+*^ mutants and cells were selected with 40μg/ml of G418 (Geneticin; Sigma-Aldrich). After inactivation of the second *RAD50* genomic allele with the *PURO* cassette, attempts to lose the Psp72-α-*NEO*-α-*RAD50* construct were performed by removing the G418 drug pressure up to 55 passages.

### DNA electrophoresis

Genomic DNAs from clones were isolated using DNAzol as recommended by the manufacturer (Invitrogen). SacI or Sau3aI/AluI/RsaI digested genomic DNAs or separated chromosomes were subjected to Southern blot hybridization with [α-32P] dCTP-labeled DNA probes according to standard protocols [96]. All probes were obtained by PCR from *L*. *infantum* genomic DNAs except the telomeric probe obtained from a Psp72-PT4 [97]. Intact chromosomes were prepared from *L*. *infantum* promastigotes harvested from log phase cultures, washed once in 1X Hepes-NaCl buffer then lysed in situ in 1% low melting agarose plugs as described in [38]. *Leishmania* intact chromosomes were separated in 1X TBE buffer (from 10X TBE: 1M Tris, 1M Acid boric, 0,02M EDTA) by Pulsed-Field Gel Electrophoresis (PFGE) using a Bio-Rad CHEF-DRIII apparatus at 5V/cm and a 120° separation angle as described previously [47]. The range of chromosome separation was between 150 and 1500 kb.

### DNA preparation for next-generation sequencing

Late log phase promastigotes (30ml) were pelleted at 3000 rpm for 5 minutes and pellets were washed once with 1X HEPES-NaCl, resuspended in suspension buffer (100mM EDTA, 100mM NaCl, 10mM Tris pH 8.0), then lysed in 1% SDS and 50μg/ml proteinase K at 37°C for 2 hours. Genomic DNA was extracted with 1 volume phenol, precipitated with 2 volumes 99% ethanol, washed with 70% ethanol twice and dissolved in 1ml 1X TE buffer. RNAse A (Qiagen) was added at 20μg/ml and DNA was incubated at 37°C for 30 minutes, followed by the addition of 50μg/ml of proteinase K and 0.1% SDS at 37°C for 30 minutes. DNA was extracted with 1 volume of phenol, precipitated and washed in ethanol, and dissolved in DNase free-water (Millipore) at 37°C overnight. Sequencing libraries were produced with the Nextera DNA sample preparation kit (Illumina Inc) according to manufacturer’s instructions.

### Bioinformatics analysis and primers design

Genome sequences were determined by Illumina HiSeq 2500 101-nucleotides paired-end sequencing. Reads from each strain were aligned to the reference genome *Leishmania infantum* JPCM5 (TriTrypDB version 8.0) using Burrows-Wheeler Alignment (bwa-mem) [51] with default parameters. The maximum number of mismatches was 4, the seed length was 32 and 2 mismatches were allowed within the seed. Several python and bash scripts were created for the detection of copy number variations. Briefly, chromosomes were divided into genomic windows of 5 kb and the number of reads mapping to each windows determined and normalized to the total number of reads before inter-strains comparisons. Alignments were also performed using the Lumpy-sv and Delly software [41,52] with default parameters for split-reads alignments and discordant read pairs and only translocations found with both software were kept for validation. PCR amplification of the new junction created in the translocation was performed using primers within 750 nucleotides from the translocation breakpoint on each involved chromosome. Optimal primer length was 20 nucleotides and optimal melting temperature (Tm) was 55°C. Primer sequences are presented in S1 Table.

### Transformation efficiency

Digestion of the Psp72-α-*ZEO*-α plasmid [98] using PciI and XbaI enzymes was performed and the isolated α-*ZEO*-α fragment was used to target the alpha-tubulin loci in order to monitor the integration efficiency. Briefly, 2x10^6^ cells from WT, *MRE11*^*-/-*^ and *MRE11*^*-/-*^*RAD50*^*-/-*^ strains were transfected with 5μg of the linear α-*ZEO*-α construct. After 24h following electroporation, cells were plated on SDM-Agar plates (1%) containing Zeocin (Invitrogen) at 1mg/ml. All strains were also transfected with the plasmid Psp72-α-*ZEO*-α and with sterile water as controls. Colonies were counted after 10–15 days of plating.

### Quantitative real-time RT-PCR

RNAs were extracted using RNeasy plus mini kit (Sigma) according to the manufacturer recommendations. The cDNA was synthesized using Oligo dT_12-18_ and SuperScript II RNase H-Reverse Transcriptase (Invitrogen) and amplified in SYBR Green Supermix (Bio-Rad) using a rotator thermocycler Rotor Gene (RG 3000, Corbett Research). The expression level was derived from three technical and three biological replicates and was normalized to constitutively expressed mRNA encoding glyceraldehyde-3-phosphate dehygrogenase (*GAPDH*, *LinJ*.*36*.*2480*). The sequences of the primers used in this assay are listed in S1 Table.

### Methyl methanesulfonate (MMS) assays

*L*. *infantum* WT, *RAD50*^*-/-*^ Psp-*RAD50*, *MRE11*^*-/-*^ and *MRE11*^*-/-*^*RAD50*^*-/-*^ were resuspended at a concentration of 5x10^6^ cells/ml and exposed to increasing doses of MMS (Sigma–Aldrich). Cells were counted after 72h and reported in survival rate.

### ATPase assays

Reactions (10 μl) contained 40nM of *Leishmania infantum* RAD50 or RAD50^K42A^ (purified by double affinity purification accordingly to [99]) in 50mM Tris-HCl pH 7.5, 1mM Mg(CH_3_COO)_2_, 1mM DTT and 100 μg/ml BSA supplemented with 50 nCi [^ɣ-32^P]ATP (3000 Ci/mmole; Perkin Elmer Life Sciences). Aliquots (5 μl) were removed, stopped by addition of EDTA, and the percentage of ATP hydrolyzed was determined by thin layer chromatography followed by quantification using a Fujifilm Phosphoimager.

### Data availability

The data set supporting the results of this article is available at the EMBL-EBI European Nucleotide Archive (http://www.ebi.ac.uk/ena) under study accession number PRJEB11440 with sample accessions ERS934506, ERS934507 and ERS934508 for *L*. *infantum MRE11*^*-/-*^*RAD50*^*-/-*^, *L*. *infantum MRE11*^*-/-*^ and *L*. *infantum* JPCM5, respectively. *L*. *infantum* 263 WT sequencing data is available under the study ERP001815 and sample accession number ERS179382.

## Supporting Information

S1 Fig *RAD50* gene conditional inactivation in *L*. *infantum*. **(A)** Schematic representation of the *RAD50* locus in *L*. *infantum* before and after integration of the inactivation cassettes blasticidin-S deaminase (5’-*BLAST-3’*), puromycin acetyltransferase (5’-*PURO-3’*). S, SacI restriction sites. **(B)** Southern blot analysis with genomic DNAs digested with SacI were hybridized with probes covering the 5’ flanking region of *RAD50*. Lanes: 1, *L*.*infantum* WT; 2, *PURO RAD50*^*-/+*^; 3, *BLAST RAD50*^*-/+*^; 4, *PURO*/*BLAST*/WT *RAD50*^*-/-/+*^. **(C)** *RAD50* mRNA levels were analyzed by quantitative real-time RT-PCR. The *RAD50* RNA expression ratios were normalized to *GAPDH* expression. 1, *L*.*infantum* WT; 2, *PURO RAD50*^*-/+*^; 3, *BLAST RAD50*^*-/+*^; 4, *PURO*/*BLAST*/WT *RAD50*^*-/-/+*^; 5, *MRE11*^*-/-*^; 6, *MRE11*^*-/-*^*RAD50*^*-/-*^.
(TIF)

S2 Fig *RAD50* gene inactivation in *L*. *infantum* in the presence of an episomal *RAD50*^*K42A*^. **(A)** Schematic representation of the *RAD50* locus in *L*. *infantum* before and after integration of the inactivation cassettes blasticidin-S deaminase (5’-*BLAST-3’*), puromycin acetyltransferase (5’-*PURO-3’*) and transfection construct Psp-*NEO*-*RAD50*^*K42A*^. S, SacI restriction sites. **(B, C)** Southern blot analysis with genomic DNAs digested with SacI were hybridized with probes covering either the 5’ flanking region of *RAD50* **(B)** or the *RAD50* ORF **(C).** Lanes: 1, *L*.*infantum* WT; 2, WT Psp-*NEO-RAD50*; 3, WT Psp-*NEO*-*RAD50*^*K42A*^; 4, *RAD50*^*-/-*^ Psp-*NEO*-*RAD50*; 5, *RAD50*^*-/-*^ Psp-*NEO*-*RAD50*^*K42A*^. **(D)** Purified LiRAD50 and LiRAD50^K42A^ proteins (300 ng) were loaded on an 8% SDS-PAGE, run then stained with Coomassie blue (left panel). Percentage of ATP hydrolysis was measured for both LiRAD50 and LiRAD50^K42A^ (40nM).
(TIF)

S3 Fig *RAD50* gene inactivation in MRE11^H210Y^ nuclease dead *L*. *infantum* parasites. **(A)** Schematic representation of the *RAD50* locus in *L*. *infantum* before and after integration of the inactivation cassettes blasticidin-S deaminase (5’-*BLAST-3’*) and neomycin phosphotransferase (5’-*NEO-3’*). S, SacI restriction sites. **(B, C)** Southern blot analysis with genomic DNAs digested with SacI were hybridized with probes covering either the 5’ flanking region of *RAD50* **(B)** or the *RAD50* ORF **(C).** Lanes: 1, *L*.*infantum* WT; 2, *HYG/PUR-MRE11*^*H210Y*^; 3, *HYG/PUR-MRE11*^*H210Y*^ *BLAST/NEO/*WT *RAD50*^*-/-/+*^.
(TIF)

S4 Fig Phenotypic analysis of *MRE11* and *RAD50* inactivation. **(A, C)** Growth retardation of promastigote *MRE11* and *RAD50* null mutants. **(B, D)** Susceptibility to methyl methanesulfonate (MMS). *L*. *infantum* WT (○), *RAD50*^*-/-*^ Psp-*NEO*-*RAD50* (●), *MRE11*^*-/-*^ (□), *MRE11*^*-/-*^*RAD50*^*-/-*^ (■), WT Psp-*RAD50* (▲), *MRE11*^*-/-*^ Psp-*RAD50* (▼). **(E)** Inactivation of *LiMRE11* impairs gene targeting with a *ZEO* inactivation cassette.
(TIF)

S5 Fig Pulse-field gels of MTX-resistant clones derived from the *MRE11*^*-/-*^*RAD50*^*-/-*^. *L*. *infantum* chromosomes were separated by pulse-field gel electrophoresis using a separation range between 150 kb and 1500 kb and incubated with ethidium bromide. No bands similar to linear amplicons could be observed.
(TIF)

S6 Fig Telomeric exchange between chromosome 27 and chromosome 02 in *L*. *infantum* WT 263. **(A)** Schematic representation of the fusion between chromosomes 27 telomeric and chromosome 02 subtelomeric regions. **(B)** DNA sequences obtained from direct sequencing of the junction 27–02. Insertion of 21 bp between sequences of chromosome 27 and chromosome 02 is indicated in bold.
(TIF)

S7 Fig Translocation breakpoints and internal duplications on chromosomes 18 and 20. Log_2_-transformed normalized read counts for non-overlapping 5 kb genomic windows on chromosomes 18 **(A)** and 20 **(B)**. Arrows indicate direction and breakpoints of the translocations. Asterik indicates internal duplications on chromosomes 18 and 20 present in T 18–20. Blue, *L*. *infantum* 263 WT; orange, *LiMRE11*^*-/-*^ and green, *LiMRE11*^*-/-*^*RAD50*^*-/-*^.
(TIF)

S8 Fig Reduction of mapped read counts at subtelomeric locations in *L*. *infantum MRE11*^*-/-*^*RAD50*^*-/-*^ cells. Log_2_-transformed normalized read counts on chromosomes 09, 15, 20, 23, 24, 31, 32 and 36.
(TIF)

S9 Fig Alterations in telomeric sequences in the *MRE11*^*-/-*^*RAD50*^*-/-*^ null mutant. Genomic DNAs of WT, *MRE11*^*-/-*^ and *MRE11*^*-/-*^*RAD50*^*-/-*^ cells were isolated, digested with Sau3aI/AluI/RsaI as described in [61] and hybridized with a telomeric probe **(A)** and a *PTR1* probe **(B)**. Lanes: 1, L.infantum WT; 2, *MRE11*^*-/-*^ and 3, *MRE11*^*-/-*^*RAD50*^*-/-*^.
(TIF)

S1 Table Primers used in this study were designed using PrimerQuest software. (DOCX)
