# Supplementary figures and images for: Chromosomal Translocations in the Parasite Leishmania by a MRE11/RAD50-Independent Microhomology-Mediated End Joining Mechanism
Source: PLoS Genet. 2016 Jun 17;12(6):e1006117. doi: 10.1371/journal.pgen.1006117 (PMC4912120; doi:10.1371/journal.pgen.1006117)

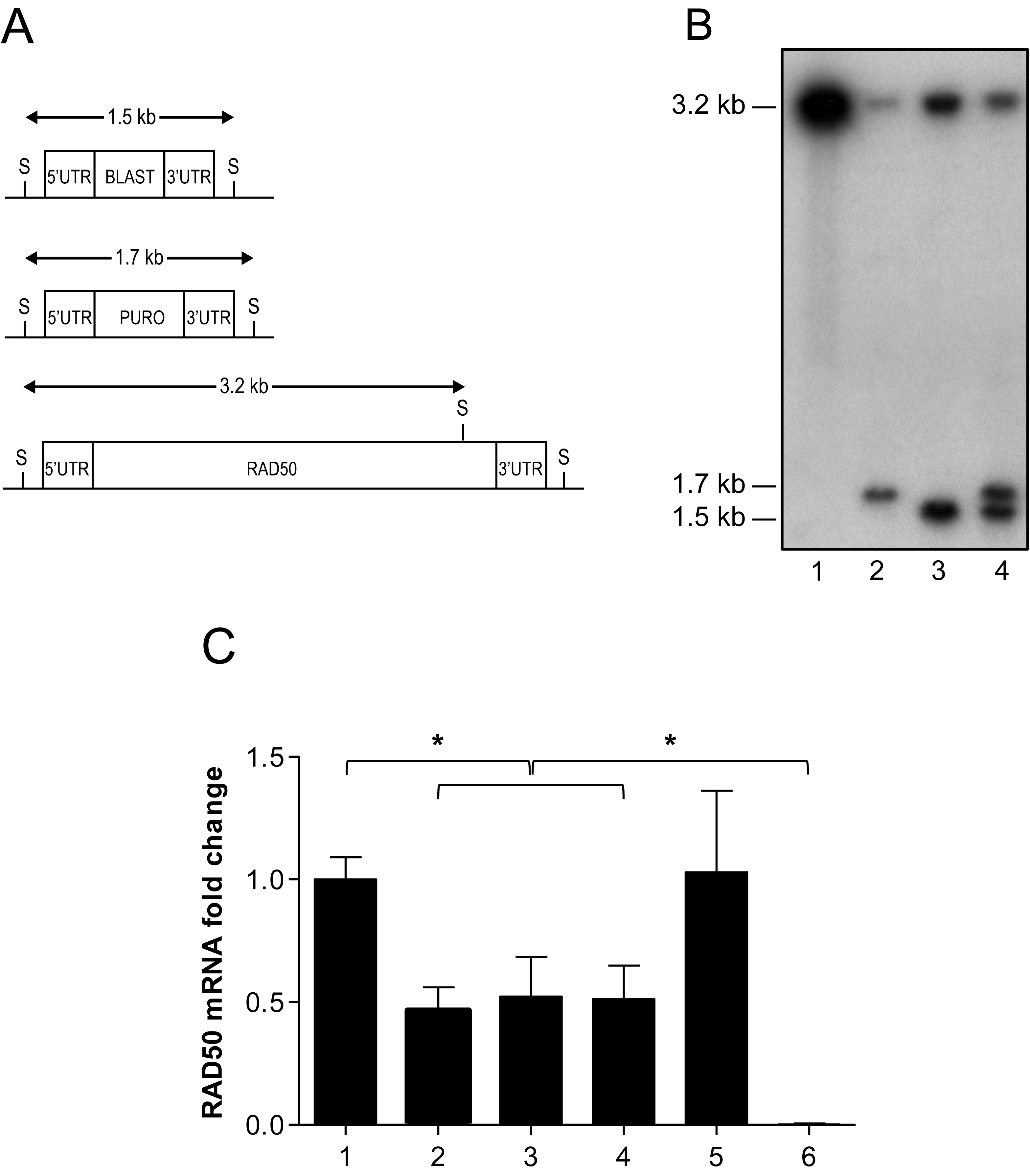

Supplement: S1 Fig — (A) Schematic representation of the RAD50 locus in L. infantum before and after integration of the inactivation cassettes blasticidin-S deaminase (5’-BLAST-3’), puromycin acetyltransferase (5’-PURO-3’). S, SacI restriction sites. (B) Southern blot analysis with genomic DNAs digested with SacI were hybridized with probes covering the 5’ flanking region of RAD50. Lanes: 1, L.infantum WT; 2, PURO RAD50-/+; 3, BLAST RAD50-/+; 4, PURO/BLAST/WT RAD50-/-/+. (C) RAD50 mRNA levels were analyzed by quantitative real-time RT-PCR. The RAD50 RNA expression ratios were normalized to GAPDH expression. 1, L.infantum WT; 2, PURO RAD50-/+; 3, BLAST RAD50-/+; 4, PURO/BLAST/WT RAD50-/-/+; 5, MRE11-/-; 6, MRE11-/-RAD50-/-. (TIF) [file pgen.1006117.s001.tif]

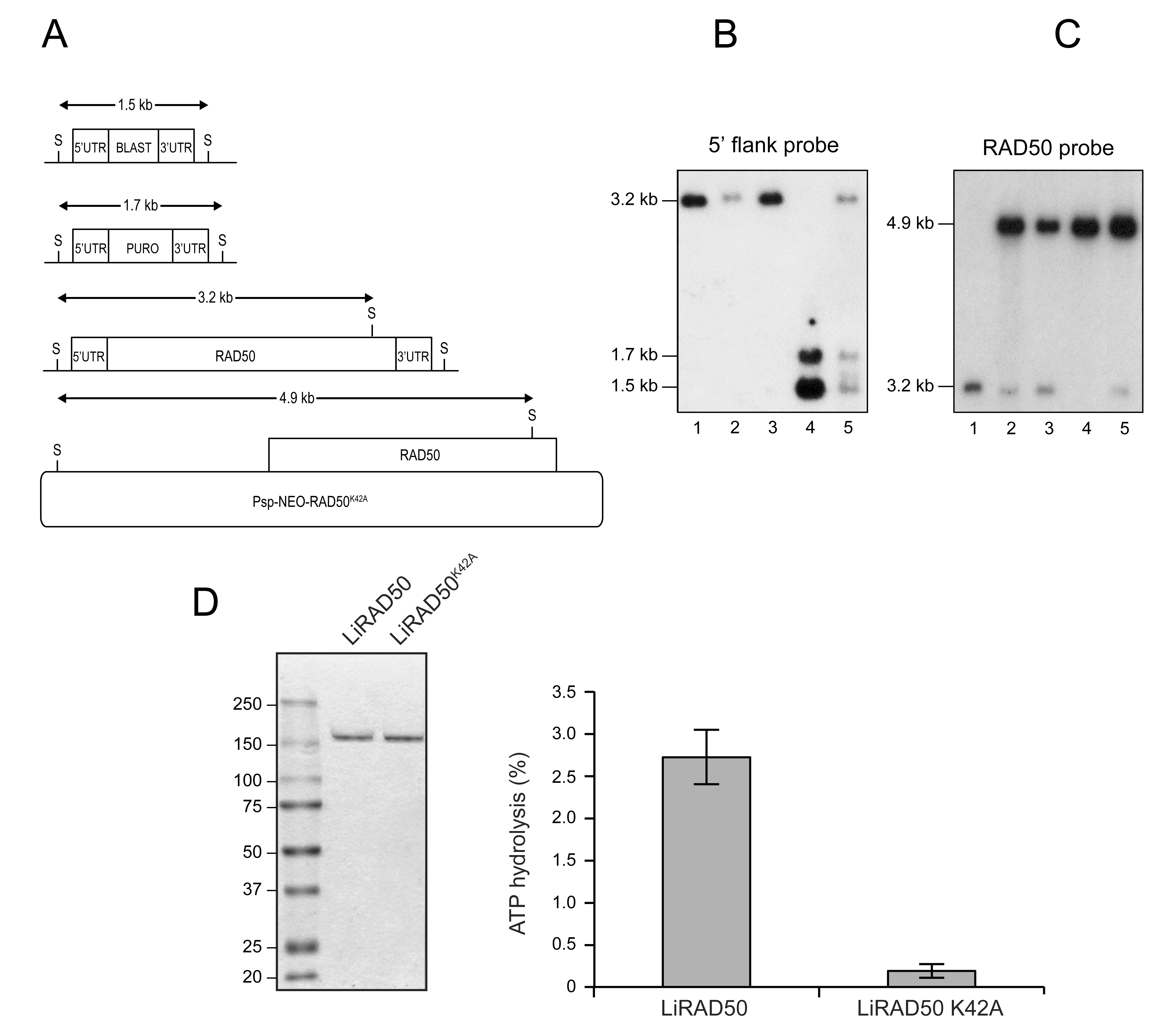

Supplement: S2 Fig — (A) Schematic representation of the RAD50 locus in L. infantum before and after integration of the inactivation cassettes blasticidin-S deaminase (5’-BLAST-3’), puromycin acetyltransferase (5’-PURO-3’) and transfection construct Psp-NEO-RAD50K42A. S, SacI restriction sites. (B, C) Southern blot analysis with genomic DNAs digested with SacI were hybridized with probes covering either the 5’ flanking region of RAD50 (B) or the RAD50 ORF (C). Lanes: 1, L.infantum WT; 2, WT Psp-NEO-RAD50; 3, WT Psp-NEO-RAD50K42A; 4, RAD50-/- Psp-NEO-RAD50; 5, RAD50-/- Psp-NEO-RAD50K42A. (D) Purified LiRAD50 and LiRAD50K42A proteins (300 ng) were loaded on an 8% SDS-PAGE, run then stained with Coomassie blue (left panel). Percentage of ATP hydrolysis was measured for both LiRAD50 and LiRAD50K42A (40nM). (TIF) [file pgen.1006117.s002.tif]

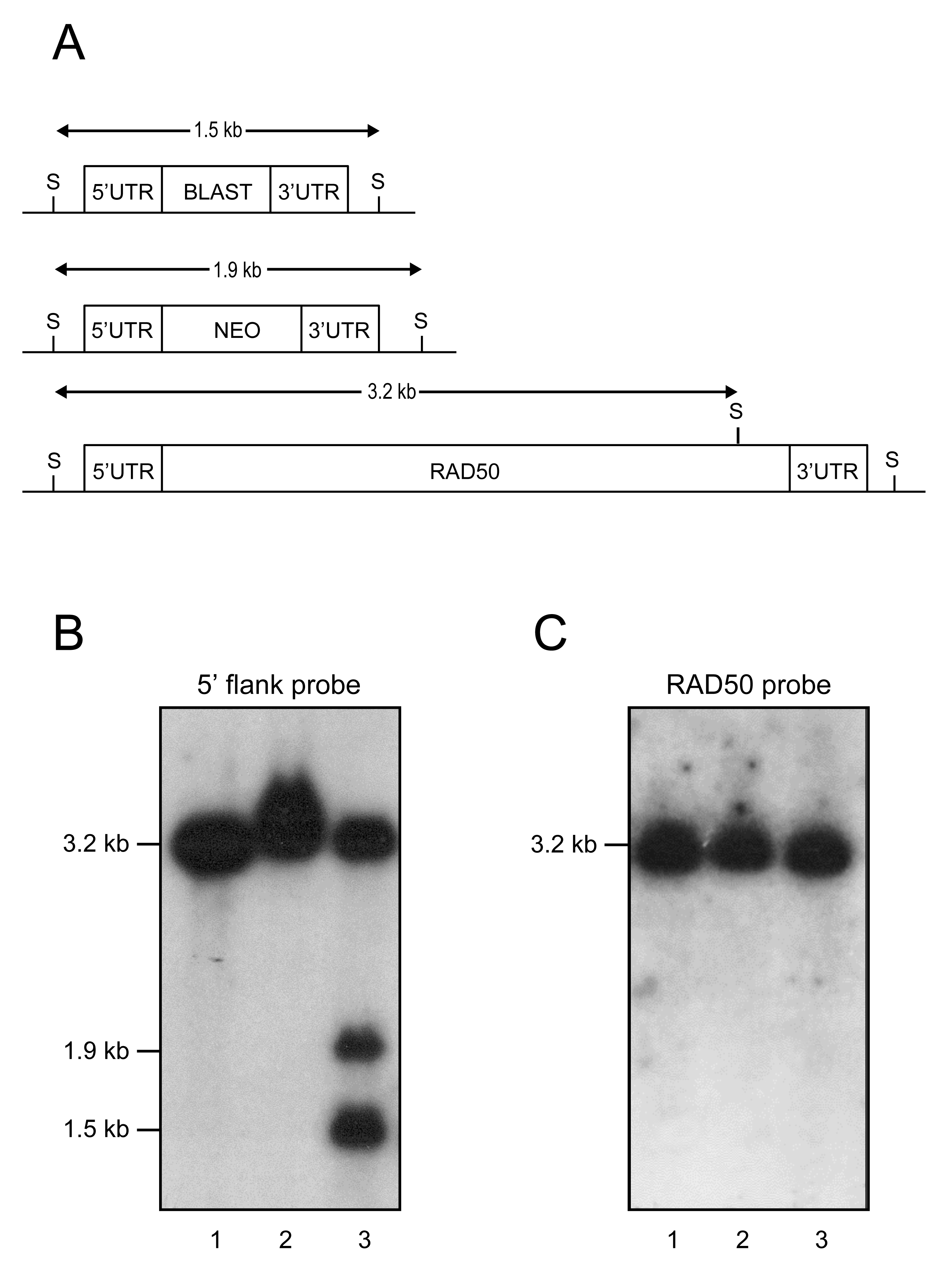

Supplement: S3 Fig — (A) Schematic representation of the RAD50 locus in L. infantum before and after integration of the inactivation cassettes blasticidin-S deaminase (5’-BLAST-3’) and neomycin phosphotransferase (5’-NEO-3’). S, SacI restriction sites. (B, C) Southern blot analysis with genomic DNAs digested with SacI were hybridized with probes covering either the 5’ flanking region of RAD50 (B) or the RAD50 ORF (C). Lanes: 1, L.infantum WT; 2, HYG/PUR-MRE11H210Y; 3, HYG/PUR-MRE11H210Y BLAST/NEO/WT RAD50-/-/+. (TIF) [file pgen.1006117.s003.tif]

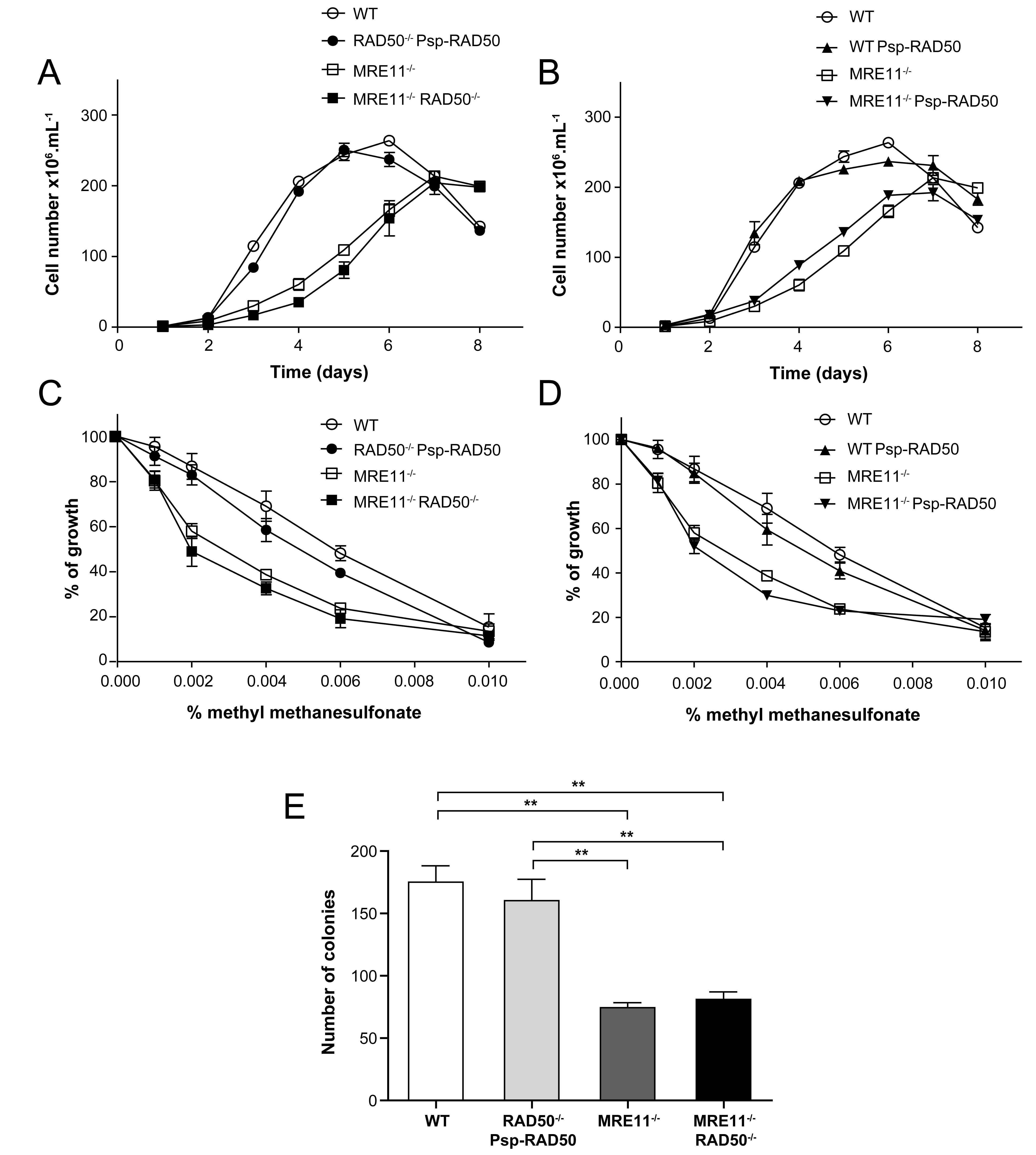

Supplement: S4 Fig — (A, C) Growth retardation of promastigote MRE11 and RAD50 null mutants. (B, D) Susceptibility to methyl methanesulfonate (MMS). L. infantum WT (○), RAD50-/- Psp-NEO-RAD50 (●), MRE11-/- (□), MRE11-/-RAD50-/- (■), WT Psp-RAD50 (▲), MRE11-/- Psp-RAD50 (▼). (E) Inactivation of LiMRE11 impairs gene targeting with a ZEO inactivation cassette. (TIF) [file pgen.1006117.s004.tif]

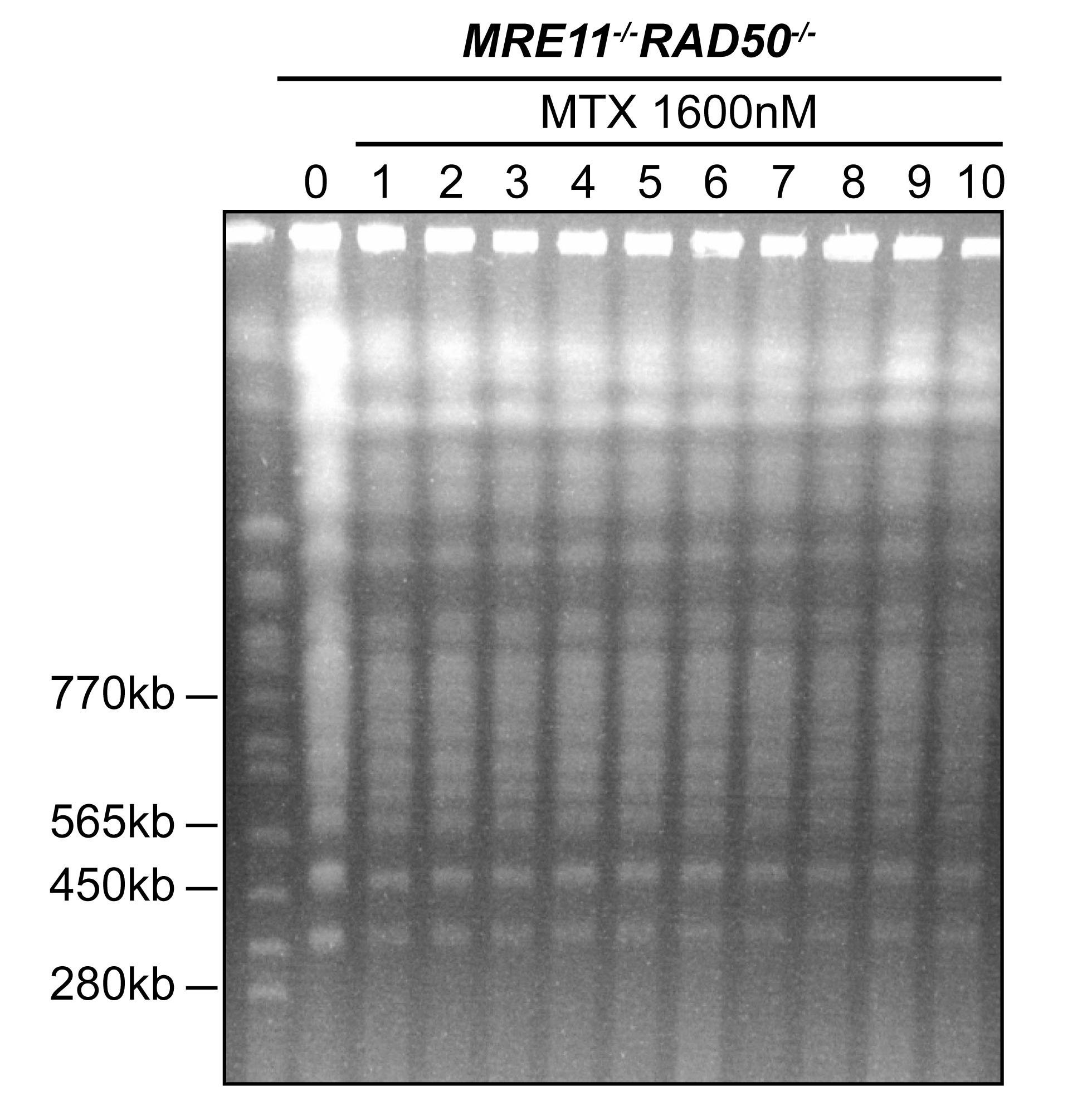

Supplement: S5 Fig — L. infantum chromosomes were separated by pulse-field gel electrophoresis using a separation range between 150 kb and 1500 kb and incubated with ethidium bromide. No bands similar to linear amplicons could be observed. (TIF) [file pgen.1006117.s005.tif]

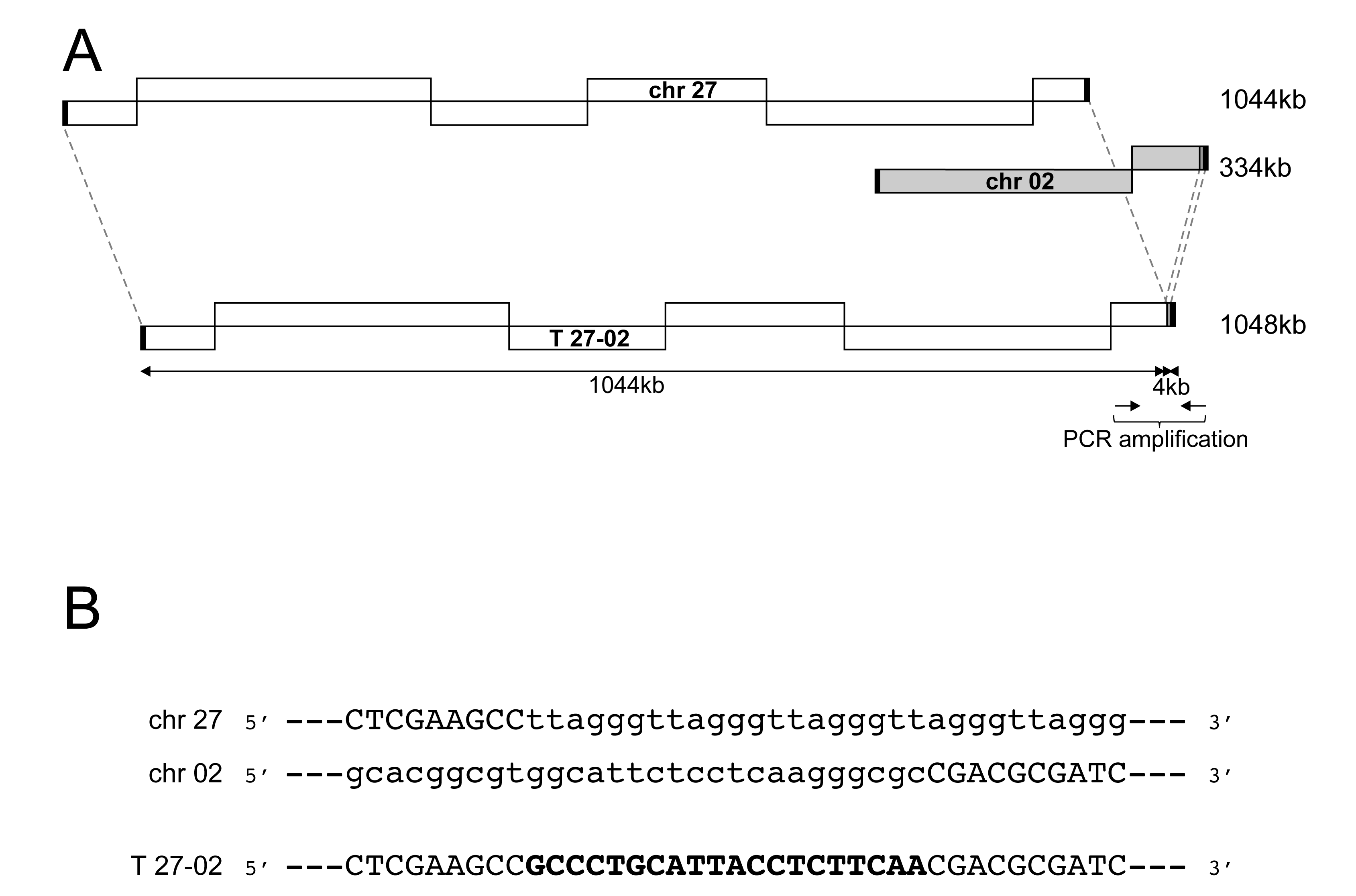

Supplement: S6 Fig — (A) Schematic representation of the fusion between chromosomes 27 telomeric and chromosome 02 subtelomeric regions. (B) DNA sequences obtained from direct sequencing of the junction 27–02. Insertion of 21 bp between sequences of chromosome 27 and chromosome 02 is indicated in bold. (TIF) [file pgen.1006117.s006.tif]

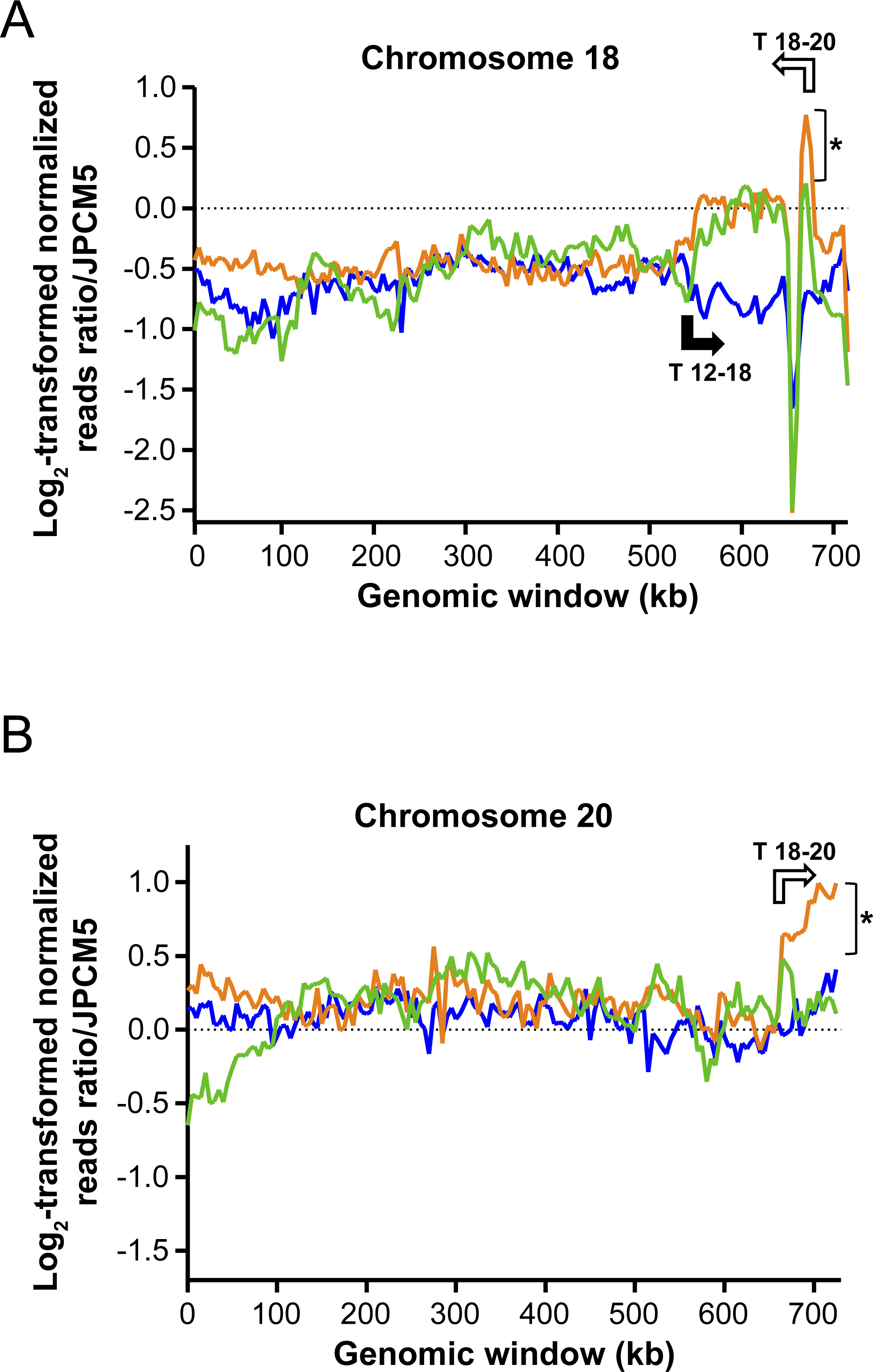

Supplement: S7 Fig — Log2-transformed normalized read counts for non-overlapping 5 kb genomic windows on chromosomes 18 (A) and 20 (B). Arrows indicate direction and breakpoints of the translocations. Asterik indicates internal duplications on chromosomes 18 and 20 present in T 18–20. Blue, L. infantum 263 WT; orange, LiMRE11-/- and green, LiMRE11-/-RAD50-/-. (TIF) [file pgen.1006117.s007.tif]

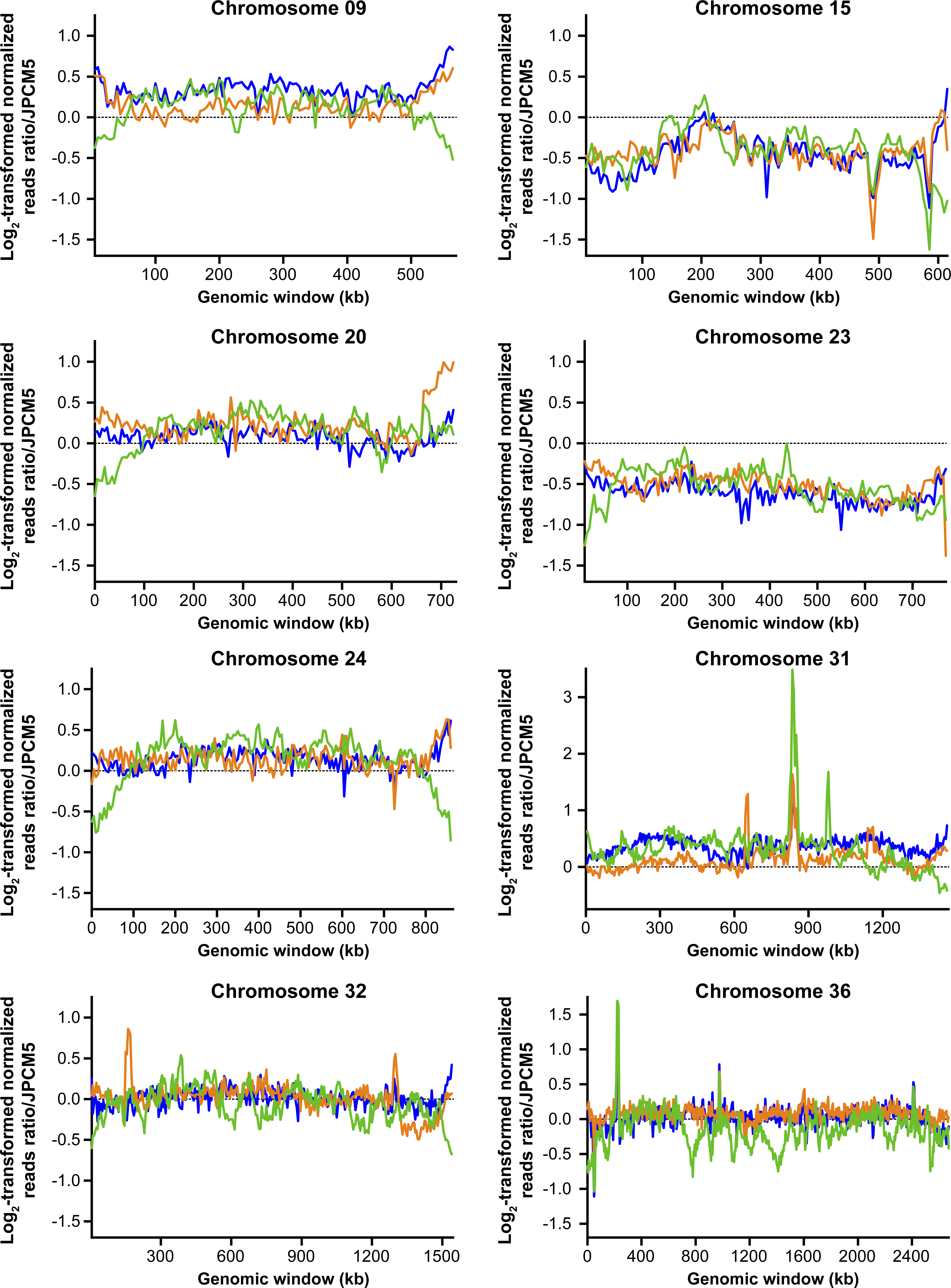

Supplement: S8 Fig — Log2-transformed normalized read counts on chromosomes 09, 15, 20, 23, 24, 31, 32 and 36. (TIF) [file pgen.1006117.s008.tif]

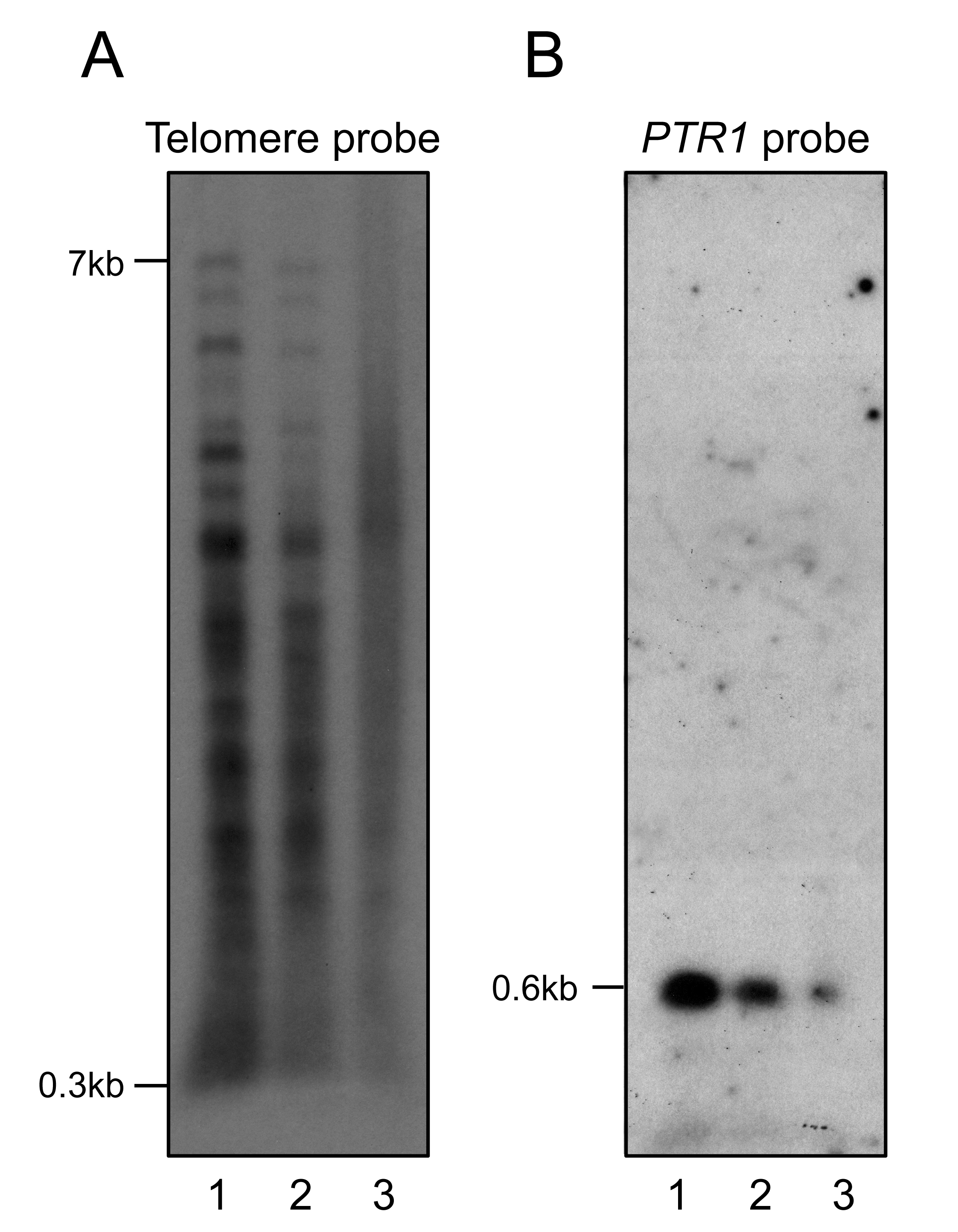

Supplement: S9 Fig — Genomic DNAs of WT, MRE11-/- and MRE11-/-RAD50-/- cells were isolated, digested with Sau3aI/AluI/RsaI as described in [61] and hybridized with a telomeric probe (A) and a PTR1 probe (B). Lanes: 1, L.infantum WT; 2, MRE11-/- and 3, MRE11-/-RAD50-/-. (TIF) [file pgen.1006117.s009.tif]
