# Supplementary material for: Chromosomal Translocations in the Parasite Leishmania by a MRE11/RAD50-Independent Microhomology-Mediated End Joining Mechanism
Source: PLoS Genet. 2016 Jun 17;12(6):e1006117. doi: 10.1371/journal.pgen.1006117 (PMC4912120; doi:10.1371/journal.pgen.1006117)
Supplement: S1 Table — (DOCX) [file pgen.1006117.s010.docx]

**Table S1.** Primers used in this study.

| **Primer name** | **Sequence (5'-3')** |
| --- | --- |
| a | CGGCCAACGAACCTCTGGCA |
| a' | CCGAGGTGGTTGTCGGTCGT |
| b | GCGAGAGTTTGGTGACGAGG |
| b' | AACTTCTCGATGCTCGTCAT |
| C | GCGAGAGTTTGGTGACGAGG |
| D | TGAGACAAAGGCTTGGCCATCACCTAGATGCAAAGGAAAA |
| E | GTGGGCTTGTACTCGGTCATCACCTAGATGCAAAGGAAAA |
| F | AATCCATCTTGTTCAATCATCACCTAGATGCAAAGGAAAA |
| G | ATGGCCAAGCCTTTGTCTCA |
| H | ACAGAACTGTCTCTGCTGCCTTAGCCCTCCCACACATAAC |
| I | ATGACCGAGTACAAGCCCAC |
| J | ACAGAACTGTCTCTGCTGCCTCAGGCACCGGGCTTGCGGG |
| K | ATGATTGAACAAGATGGATT |
| L | ACAGAACTGTCTCTGCTGCCTCAGAAGAACTCGTCAAGAAG |
| M | GGCAGCAGAGACAGTTCTGT |
| N | AGCACACGCCGGGAGACAGG |
| O | ATGACGAGCATCGAGAAGTT |
| P | TCACGCAAAAAGCTGGTCAA |
| Q | GGCAAGAATGGTGCTGGCGCAACCACCATCATCGAGGCGC |
| R | GCGCCTCGATGATGGTGGTTGCGCCAGCACCATTCTTGCC |
| T12-06 forward | GGGCTGCGATGTCTTCATTA |
| T12-06 reverse | CACGTCTTCGTCGAGCTTAC |
| T12-18 forward | TGATTCACACTTGCCCTTAC |
| T12-18 reverse | CCAGTAAGGCAGTGCGTGGG |
| T18-20 forward | TTGCCTTCGGCTTCCATATC |
| T18-20 reverse | CAAACATGCTGTCGTTGGAAG |
| T08-17 forward | GGGCGGTGTGCGTCCCTCTC |
| T08-17 reverse | ACAGTGTGCGGCATCGTCGG |
| T27-02 forward | CTCATCATCTCCACCGTCAC |
| T27-02 reverse | GCTTCGCCCACGCAATCGTC |
| RAD50 qRT-PCR forward | CCGAAGAGTTGCGGAAGAAA |
| RAD50 qRT-PCR reverse | CAGTTGAGCCACGGTACTATTC |
| GAPDH qRT-PCR forward | GTACACGGTGGAGGCTGTG |
| GAPDH qRT-PCR reverse | CCCTTGATGTGGCCCTCGG |
